# Whole‐Body Pattern of Muscle Degeneration and Progression in Sarcoglycanopathies

**DOI:** 10.1002/acn3.70303

**Published:** 2025-12-31

**Authors:** Laura Costa‐Comellas, Mauro Monforte, Angel Sanchez‐Montañez, Penélope Romero‐Duque, Elena Pegoraro, Jordi Díaz‐Manera, Dmitry Vlodavets, Lorenzo Maggi, Marco Moscatelli, Adele D‘Amico, Montse Olivé, Jorge Alonso‐Pérez, Giacomo Comi, José Miguel Escudero‐Fernández, Gabriela S. Urcuyo, Anna Pichiecchio, Angela Berardinelli, Kristl G. Claeys, Claudio Bruno, Chiara Panicucci, Sara Bortolani, Eleonora Torchia, Enzo Ricci, Soledad Monges, Jorge A. Bevilacqua, Jorge Diaz‐Jara, Maggie C. Walter, Simone Thiele, Nicoline Løkken, John Vissing, Susana Quijano‐Roy, Robert Y. Carlier, Nicol C. Voermans, Chiara Marini‐Bettolo, Michela Guglieri, Volker Straub, Lea Leonardis, Francina Munell, David Gómez‐Andrés, Giorgio Tasca

**Affiliations:** ^1^ Pediatric Neuromuscular Disorders Unit, Pediatric Neurology Department Vall D‘hebron Barcelona Hospital Campus Barcelona Spain; ^2^ Department of Pediatrics Autonomous University of Barcelona Barcelona Spain; ^3^ Pediatric Neurology Research Group Vall D‘hebron Research Institute (VHIR) Barcelona Spain; ^4^ Unità Operativa Complessa di Neurologia Fondazione Policlinico Universitario A. Gemelli IRCCS Rome Italy; ^5^ Pediatric Neuroradiology Unit, Radiology Department Vall D‘hebron University Hospital Barcelona Spain; ^6^ Department of Neurological and Psychiatric Sciences University of Padova Padova Italy; ^7^ John Walton Muscular Dystrophy Research Centre, NIHR Newcastle Biomedical Research Centre Newcastle University and Newcastle Hospitals NHS Foundation Trust Newcastle Upon Tyne UK; ^8^ Neuromuscular Diseases Unit Foundation of the Hospital de la Santa Creu i Sant Pau Research Institute Barcelona Spain; ^9^ Biomedical Network Research Centre on Rare Diseases (CIBERER) Madrid Spain; ^10^ Russian Children Neuromuscular Center, Veltischev Clinical Pediatrics and Pediatric Surgery Research Institute of Pirogov Russian National Research Medical University Moscow Russia; ^11^ Neuroimmunology and Neuromuscular Disease Unit Fondazione IRCCS Istituto Carlo Besta Milano Italy; ^12^ Neuroradiology Unit Fondazione IRCCS Istituto Carlo Besta Milano Italy; ^13^ Neuromuscular and Neurodegenerative Disorders Unit, Department of Neurosciences Bambino Gesu‘ Children‘s Hospital IRCCS Roma Italy; ^14^ Neuromuscular Diseases Unit, Department of Neurology Hospital de la Santa Creu i Sant Pau Barcelona Spain; ^15^ Neuromuscular Disorders Unit, Neurology Department Hospital Universitario Nuestra Senora de la Candelaria Santa Cruz de Tenerife Spain; ^16^ Fundación Canaria Instituto de Investigación Sanitaria de Canarias Las Palmas de Gran Canaria Spain; ^17^ Neurology Unit Fondazione IRCCS ca‘ Granda Ospedale Maggiore Policlinico Milan Italy; ^18^ Department of Pathophysiology and Transplantation (DEPT) Dino Ferrari Centre Milan Italy; ^19^ University of Milan Milan Italy; ^20^ Department of Brain and Behavioural Sciences University of Pavia Pavia Italy; ^21^ Neuroradiology Department, Advanced Imaging Center IRCCS Mondino Foundation Pavia Italy; ^22^ Child Neurology and Psychiatry Unit RCCS Mondino Foundation Pavia Italy; ^23^ Department of Neurology, University Hospitals Leuven and Laboratory for Muscle Diseases and Neuropathies, Department of Neurosciences KU Leuven Leuven Belgium; ^24^ Center of Translational and Experimental Myology IRCCS Istituto Giannina Gaslini Genova Italy; ^25^ Department of Neuroscience, Rehabilitation, Ophthalmology, Genetics, Maternal and Child Health (DINOGMI) University of Genova Genova Italy; ^26^ Institute of Neuroscience Università Cattolica del Sacro Cuore (UCSC) Rome Italy; ^27^ Department of Neurology, “Prof. Dr. Juan P. Garrahan” Pediatric Hospital Combate de los Pozos Buenos Aires Argentina; ^28^ Neuromuscular Unit, Neurology and Neurosurgery Department University of Chile Clinical Hospital Santiago de Chile Chile; ^29^ Neurology and Neurosurgery Department Clínica Dávila Santiago Chile; ^30^ Radiology Department University of Chile Clinical Hospital Santiago de Chile Chile; ^31^ Department of Neurology, Friedrich‐Baur‐Institut, LMU Hospital Ludwig‐Maximilians‐University Munich Germany; ^32^ Copenhagen Neuromuscular Center, Department of Neurology Rigshospitalet, University of Copenhagen Copenhagen Denmark; ^33^ Neuromuscular Unit, Child Neurology and ICU Department University Hospital Raymond‐Poincaré APHP, GH Université Paris‐Saclay Garches France; ^34^ University of Versailles Saint‐Quentin‐En‐Yvelines INSERM‐UVSQ Versailles France; ^35^ DMU Smart Imaging, Medical Imaging Department University Hospital Raymond‐Poincaré APHP, GH Université Paris‐Saclay Garches France; ^36^ Department of Neurology Radboud University Nijmegen the Netherlands; ^37^ Institute of Clinical Neurophysiology University Medical Centre Ljubljana Ljubljana Slovenia; ^38^ Department of Neurology, Faculty of Medicine University of Ljubljana Ljubljana Slovenia

**Keywords:** disease progression, limb girdle muscular dystrophy, muscle MRI, sarcoglycanopathy, whole‐body pattern

## Abstract

**Objective:**

To characterize whole‐body intramuscular fat distribution pattern in patients with sarcoglycanopathies and explore correlations with disease severity, duration and age at onset.

**Methods:**

Retrospective, cross‐sectional, multicentric study enrolling patients with variants in one of the four sarcoglycan genes who underwent whole‐body muscle MRI. Intramuscular fatty replacement was evaluated on T1‐weighted images and represented by heatmaps. Dimensionality reduction and linear spline models examined relationships between patterns of intramuscular fat replacement and clinical findings.

**Results:**

MRI scans from 64 patients (age range 4–67 years) covering 4160 muscles were analyzed. Disease severity ranged from asymptomatic (9%) to non‐ambulant (39%) patients. Sarcoglycanopathies showed consistent, selective patterns of muscle involvement across genotypes. Latissimus dorsi and subscapularis were the earliest affected muscles in the upper body, whereas head, neck and forearm muscles remained largely preserved. Distinct gradients characterized the topography of degeneration both within individual muscles and along body and limb axes. Disease severity correlated with MRI changes in both upper and lower body muscles, and with one of the dimensions identified by the multi‐correspondence analysis. Patients with onset in the first decade showed a steeper cross‐sectional association between disease duration and MRI abnormalities, while later‐onset patients displayed a more gradual, linear relationship.

**Interpretation:**

Sarcoglycanopathies display selective muscle vulnerability with characteristic gradients of fat replacement. Scapular girdle muscles are affected early in the disease course. Intramuscular fat correlates with functional impairment and disease duration, supporting its use as a surrogate endpoint in clinical trials. Age at onset emerges as a critical prognostic factor.

## 1 Introduction

Sarcoglycanopathies are a group of autosomal recessive (LGMDs) caused by mutations in the α, β, γ, and δ sarcoglycan genes, recently renamed as LGMDR3 (LGMD2D), LGMDR4 (LGMD2E), LGMDR5 (LGMD2C), and LGMDR6 (LGMD2F), respectively [1]. Sarcoglycan proteins are organized in a tetrameric transmembrane complex in skeletal muscle fibers, stabilizing the sarcolemma as part of the dystrophin‐glycoprotein complex as well as playing a pivotal role in signal transduction of mechanical information [2]. The close molecular relationship between them translates into overlapping clinical phenotypes and muscle pathology findings when one of the genes is mutated, often turning the exact molecular diagnosis into a challenge [3].

Clinically, sarcoglycanopathies present with limb‐girdle muscle weakness, calf hypertrophy, elevated serum creatine kinase levels and, in some cases, dilated cardiomyopathy. Disease onset occurs in a wide age range and early age at onset is an independent predictor for loss of ambulation [4, 5].

Muscle MRI is an important contributor to the diagnostic workup of muscle disorders, and increasing diagnostic accuracy is more relevant than ever with the recent emergence of specific treatments for several neuromuscular disorders [6, 7, 8, 9]. The description of muscle imaging findings in sarcoglycanopathies has been limited to the lower body (LB) and limbs, where the earliest involved muscle groups are adductors, glutei, and posterior thigh muscles, while the sartorius, gracilis, and lower leg muscles are relatively spared throughout the disease course. Another frequent feature is the proximo‐distal gradient of intramuscular fat of the anterior thigh muscles [10]. However, data about the specificity of this pattern is missing and the relationship between the global imaging phenotype and the progression of the disease has not been explored in detail. The aims of this study are: (1) to describe the pattern and degree of whole‐body (WB) muscle involvement in patients with sarcoglycanopathies, with a particular focus on the so far undescribed upper body (UB) region, and (2) to assess the relationship between intramuscular replacement by fat and disease duration and severity by combining different data analysis approaches.

## 2 Methods

### 2.1 Patients

Patients with a diagnosis of LGMDR3‐6 were eligible for the study. Criteria for a confirmed diagnosis of LGMDR3‐6 were: (a) two pathogenic or likely pathogenic variants in one of the sarcoglycan genes according to the ACMG (American College of Medical Genetics) criteria or reports in LOVD (Leiden Open Variation Database), or (b) one pathogenic variant plus a variant of uncertain significance in a sarcoglycan gene, together with reduced staining of one or more sarcoglycans on muscle tissue. These patients were recruited in 16 neuromuscular centers in Europe (Belgium, Denmark, France, Germany, Italy, the Netherlands, Slovenia, Spain, and the United Kingdom), Russia, and South America (Argentina and Chile).

Patients were enrolled if they had a muscle MRI scan which included the UB and were classified into four severity groups according to their motor status: “asymptomatic” if they had isolated hyperCKemia, “mild” if they were able to run, “intermediate” if they were ambulant but not able to run, “severe” if they were non‐ambulant or walked with support.

### 2.2 MRI Scans and Evaluation

MRI studies were performed following standard protocols, either WB [11, 12, 13] or a combination of UB and LB MRI [14, 15], using different 1.5 T or 3 T scanners, and T1‐weighted images were analyzed. All scans were reviewed for adequate field of view, symmetric positioning, and uniform signal intensity before scoring.

A total of 65 muscles on both sides were evaluated for each scan by two independent observers, a radiologist and a pediatric neurologist with experience in muscle imaging (AS and LC). In case of discrepancy between the evaluators, agreement was reached by consensus (AS, DGA and LCC). Median scores from both sides were used for analysis to further minimize possible technical bias. A four‐point modified Lamminen scale was used to evaluate signal abnormalities reflecting the extent of intramuscular fatty replacement in each muscle. Scores were defined as: 0 = normal signal intensity, 1 = slightly hyperintense, patchy intramuscular signal changes, 2 = markedly hyperintense and widespread intramuscular signal changes, 3 = total, homogeneous hyperintense signal change in whole muscle, equaling the signal intensity of the adjacent subcutaneous or paramuscular fat, or atrophy [16]. Muscles that were not adequately visualized were recorded as missing values (NA). The four‐point scoring system was chosen to increase consistency and reproducibility when assessing some of the small and flat muscles in the head, trunk, neck and arms [14]. The lower limb scans analyzed in the previous imaging study with a 5‐point scoring system were re‐scored accordingly ensuring uniformity across all body regions [10].

### 2.3 Data Analysis

Heatmaps were built to represent the hierarchical clustering of patients and muscles based on individual muscle scores [17]. Spearman's correlation coefficient was used to quantify associations between motor status and abnormal muscle signal. Muscles with significant variability in signal (homogeneity index, which is a metric that quantifies the degree of uniformity in the score of a specific muscle [18, 19], > 0.8) were selected for further analysis.

Multi‐correspondence analysis with optimal scaling [20] was applied to reduce the large set of ordinal muscle scores into a limited number of numerical dimensions capturing the main patterns of muscle involvement. Two dimensions were identified from the Scree plot and validated using 10 permuted databases (Figure S1). After assessing correlations with individual muscles and reviewing eigenvalues, the first dimension was chosen as a summary measure of overall muscle involvement. Therefore, its association with motor status at the time of MRI was assessed.

Multiple linear regression models were applied to evaluate the relationship between the first summary dimension and disease duration and how this relationship changed according to disease onset. Three types of models were used. Model 1 assumed a linear relationship between disease duration and the dimension of intramuscular fatty replacement. Based on the graphical representation of the relationship between the first summary dimension and disease duration, and the clinically recognized acceleration of functional decline during approximately the first decade of disease evolution [21], we specified a linear spline model with a knot at 10 years of disease duration. Alternative knot positions (Figure S2) were explored, and the 10‐year knot was retained as it provided the best overall performance in terms of adjusted *R* ^2^ and residual diagnostics [21]. Model 3 was built for data representation purposes and split patients into two groups depending on disease onset (patients with onset in the first decade of life or later) [4]. Models were compared using adjusted *R*. Furthermore, the relationship between the ordinal MRI signal score (0–3) for each muscle and disease duration was assessed using proportional‐odds logistic regression (ordinal logistic regression), with disease onset and its interaction with duration included as predictors. This model estimates the cumulative odds of higher grades of intramuscular fat replacement rather than a binary outcome. The relationship between signal score in individual muscle and the disease duration and the interaction of disease duration with disease onset were assessed using proportional‐odds logistic regressions [22] with results expressed as odds' ratio. Statistical analyses were performed with R (r‐project.org). The data analysis flow is displayed in Figure S3.

### 2.4 Standard Protocol Approvals, Registrations, and Patient Consents

This study was approved by the ethics committee of the Università Cattolica del Sacro Cuore (Rome, Italy; protocol 5098/14). All the collected scans were performed for diagnostic or follow‐up purposes. All involved subjects or their legal guardians gave their informed consent for MRI scans and agreed with data sharing for research purposes.

## 3 Results

### 3.1 Patients

Sixty‐four patients (43 LGMDR3, 8 LGMDR4, 12 LGMDR5, and 1 LGMDR6) were enrolled. Fifty‐seven (89%) were molecularly confirmed with two pathogenic or likely pathogenic variants *in trans* detected in one of the sarcoglycan genes (ACMG criteria or LOVD). Seven patients (11%), including two pairs of siblings, had one pathogenic variant and a second *in trans* variant of uncertain significance and a clear reduction of the staining of one or more sarcoglycan proteins on muscle tissue, which supported the diagnosis (Table S1). Fifty‐two percent of the patients underwent WB MRI, while the remaining were investigated by UB and LB MRI. Thirty‐eight of the 64 patients (59%) had been included in our previous lower‐limb imaging [10], while 26 patients (41%) were newly added. All scans and muscles were re‐scored for this new analysis.

Both sexes were similarly represented (30 females and 34 males). Patients' age ranged from 4 to 67 years (*mean* ± SD: 27 ± 17.93 years). Forty‐three patients (67%) reported symptom onset during childhood, often with difficulty in running and climbing stairs. Functional status at the time of MRI ranged widely covering the whole spectrum of the disease: six patients (9%) were asymptomatic, 14 patients (22%) were classified as mild, 19 patients (30%) were in an intermediate status, and 25 patients (39%) were classified as severely affected (Table S2).

### 3.2 Muscle Imaging Findings

#### 3.2.1 Whole‐Body Distribution of Involvement

**FIGURE 1:**
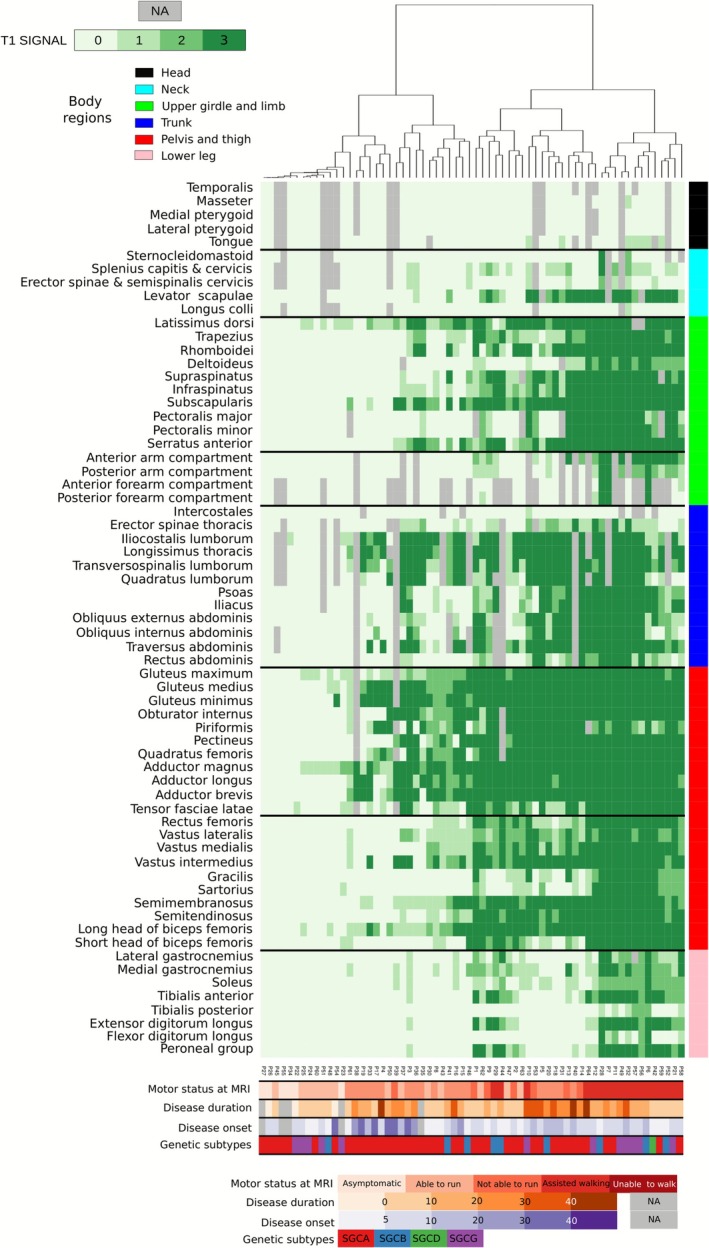
Heatmap representing signal scores in T1‐weighted MRI for each muscle in patients with sarcoglycanopathies. Muscles are displayed according to the different body regions, and patients are clustered based on the degree of similarity of muscle involvement as shown by the dendrogram. Functional status at MRI, disease duration (in years), age at disease onset (years), and mutated sarcoglycan are shown below.

A total of 4160 muscles were examined. Overall, muscle involvement was symmetric, and no clear differences were evident comparing the different disease subtypes. The heatmap representation in Figure 1 shows intramuscular fatty replacement scores in the different muscles ordered by body regions in the whole cohort, while in Figure S4 muscles are hierarchically clustered according to their degree of involvement in the different patients irrespective of the body regions.

Four categories were identified:

1. Muscles involved in most of the patients (‘positive disease pattern’), also representing the earliest and most severely affected muscles: most of them were localized in the lower body, especially in the pelvic girdle and thighs (e.g., adductor magnus, long head of biceps femoris, and gluteus maximus) and lumbar paravertebral region. From the upper body, latissimus dorsi and subscapularis muscles had a similar, early replacement pattern by fat.
2. Muscles are consistently involved only in the advanced stage, which could present a variable extent of fat replacement in the intermediate stages. Most of them were localized in the upper body (e.g., serratus anterior, trapezius, levator scapulae, infraspinatus, and supraspinatus). Muscles from the lower body, such as the short head of biceps femoris, tensor fasciae latae, vastus lateralis and medialis, rectus femoris, semitendinosus, and semimembranosus, also belonged to this group.
3. Relatively spared muscles, showing only mild to moderate fat replacement even in the severe stage. Lower leg and arm muscles, deltoid, sartorius, gracilis, rectus abdominis, and pectoralis presented a comparable sparing until advanced stages.
4. Muscles preserved even in severe stages, such as the head, neck, and forearm ones (‘negative disease pattern’).

### 3.3 Upper Body Pattern

Only nine out of 64 individuals (14%; 7 LGMDR3, 1 LGMDR4 and 1 LGMDR5) had a normal UB MRI. Most of these nine subjects were asymptomatic and their ages ranged from 4 to 47 years. Four of them also had a normal LB scan. When analyzing asymptomatic individuals, one of six showed subclinical signs of muscle involvement in the UB. This ratio increased in mild patients, with 3 abnormal UB MRIs out of 14 patients.

Cranial muscles were widely preserved even in advanced stages of the disease. The tongue showed mild involvement only in seven severely affected patients. The neck region was also well‐preserved except for the levator scapulae, involved in 29 of the 64 patients, mostly at an intermediate or severe stage. In severe stages, neck extensors exhibited low‐grade and heterogeneous involvement. The longus colli and sternocleidomastoid were always spared.

The scapular girdle was the earliest and the most affected region in the UB. Among upper‐body muscles, the latissimus dorsi was the most frequently affected in mildly symptomatic patients (score ≥ 1 in 12/14, 85%), suggesting its common involvement early in the disease course (Figure S5). Other muscles affected early in the disease were the subscapularis and serratus anterior (score ≥ 1 in 15/19, 78%, and 12/19, 63%, of the patients in intermediate stage, respectively). Supraspinatus, infraspinatus, and pectoralis muscles exhibited significant signal changes only in severe cases. The trapezius was mildly involved in intermediate stages (score 0–1 in 16/19, 84%, of the patients) and showed a more significant involvement in severe patients (score 3 in 17/25, 68%, of the patients). The deltoid muscle presented a heterogeneous involvement in advanced stages.

Arm muscles showed signs of fat replacement only in the latest disease stages, with the anterior compartment being more affected than the posterior (median score 3 vs. 1). Forearm muscles were evaluable in 49 patients and remained preserved, except in five severe patients.

Paravertebral muscles were involved in 44/64 (69%) of the scans. Lumbar paravertebral muscles were the first and most involved ones, with a median score of 2.2 for iliocostalis lumborum and longissimus thoracis ‐lumbar part‐ (also referred to as erector spinae lumborum) and a median score of 3 for transversospinalis lumborum. By contrast, thoracic paravertebral muscles (also referred to as erector spinae thoracis) were widely spared (median score 0.5) even in severe stages.

**FIGURE 2:**
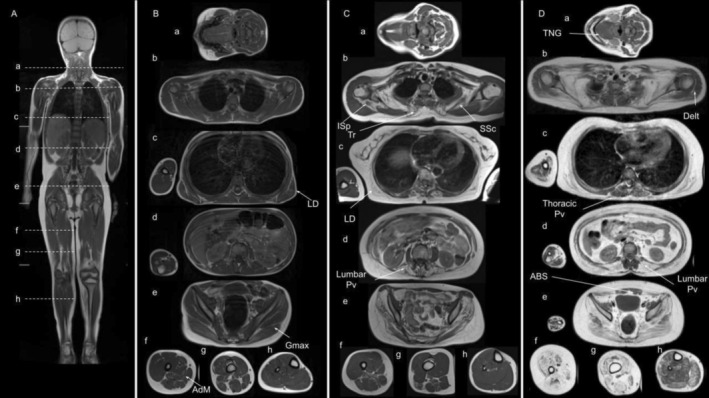
Progressive muscle involvement in sarcoglycanopathies. (A) Localizer. Coronal view of whole‐body MRI, displaying the corresponding axial slices (a–h). (B) P24, LGMDR5, mildly affected: Initial involvement of latissimus dorsi (LD), gluteus maximus (Gmax), and adductor magnus (AdM). (C) P35, LGMDR3, intermediate stage: Advanced involvement of subscapularis (SSc), infraspinatus (Isp), and trapezius (Tr) and complete replacement by fat of LD. Lumbar paravertebral muscles (Lumbar Pv) are also severely affected. (D) P1, LGMDR3, severe stage (able to walk only with support): Severe and diffuse involvement of lower paravertebral, pelvis, and thigh muscles with complete sparing of the tongue (TNG) and relative preservation of thoracic paravertebral muscles (Thoracic Pv); deltoid (Delt) and proximal arm muscles are mildly affected. Rectus abdominis (ABS) is also preserved in contrast to obliquus and transversus abdominis.

Abdominal belt muscles, particularly the rectus abdominis, were mostly preserved until advanced stages. Obliquus and transversus abdominis were always more involved than the rectus (Figure 2).

Consistent with findings reported in the lower limbs [10], characteristic intramuscular gradients of fat replacement were detected by the visual analysis of the scans. These gradients were particularly evident for some muscles and followed the body or limb axis, with relative sparing of the cranial portions of axial muscles compared to the caudal and the distal muscles and muscle portions compared to the proximal in the arms.

**FIGURE 3:**
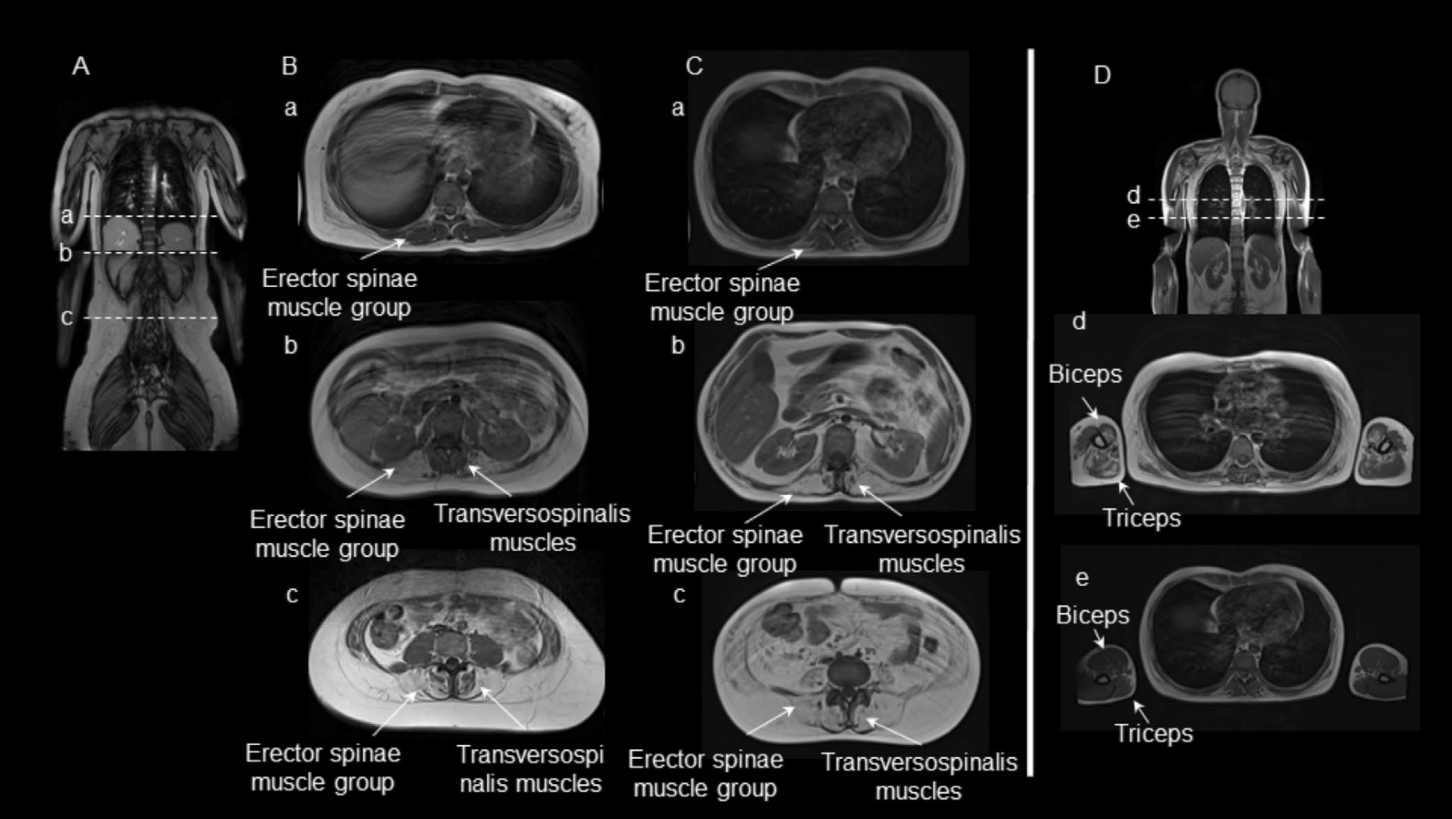
Fat replacement gradient in paraspinal and arm muscles. (A, D) Localizer images. Coronal view of WB‐MRI, displaying the corresponding axial slices (a–e). (B) P33, LGMDR3, and (C) P20, LGMDR4: Showing severe involvement of erector spinae at the lumbar level with sparing of the upper thoracic part. Transversospinalis muscles also show a similar gradient. (D) P20, LGMDR4: A proximo‐distal gradient of involvement is evident in biceps and triceps brachii muscles.

Paraspinal muscles showed a clear caudocranial fat replacement gradient maintained throughout the different degrees of severity. Most scans showed severe involvement of erector spinae and transversospinalis muscles at the lower thoracic and lumbar levels compared to a wide preservation of these muscles at higher levels (Figure 3).

**FIGURE 4:**
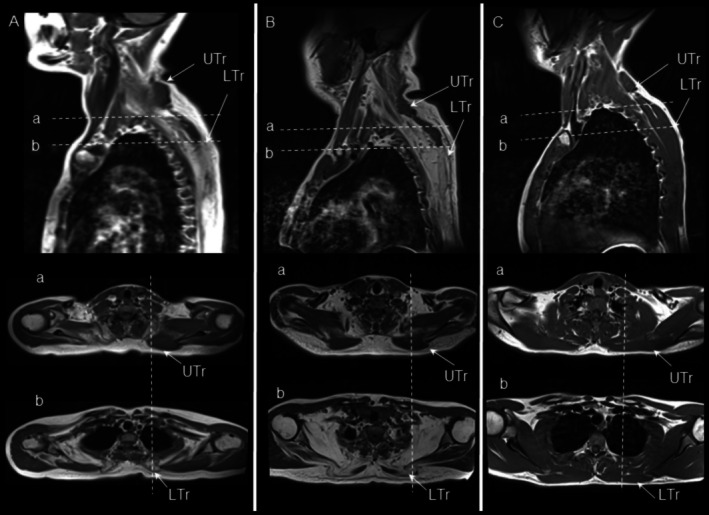
Fat replacement gradient in the trapezius muscle. (A) P35, LGMDR3, and (B) P36, LGMDR3: Show sagittal views of the trapezius muscle in two patients in an intermediate stage of the disease. The preservation of the upper trapezius (UTr) contrasts with the replacement of the lower trapezius (LTr), which can be appreciated also in the corresponding axial sections (a, b). (C) shows the same views from a healthy subject for comparison.

In addition, in 21/39 scans (54%), the lower portion of the trapezius muscle (ascending fibers) was more severely and earlier affected than the upper (descending fibers) and middle portions (transverse fibers). In the remaining scans, the trapezius showed either a homogeneous mild involvement across all portions (approximately one third of cases) or complete preservation (the remaining cases). This peculiar intramuscular pattern was more evident at mild and intermediate stages of the disease, although it could still be recognized in 28% of the most severe patients (Figure 4). Finally, seven out of 21 patients (33%) with involvement of arm muscles showed a proximal‐to‐distal gradient of signal abnormality in the biceps and triceps brachii muscles.

### 3.4 Correlation Between Clinical Features and Intramuscular Fatty Replacement

Disease severity was moderately to strongly correlated with abnormal signal scores of both UB and LB muscles (0.4 < rho < 0.8, Spearman's correlation coefficient), whereas correlations were lower in relatively spared muscles belonging to the third hierarchical clustering class (Figure S6).

To reduce the number of ordinal scores across muscles, dimension reduction technique condensed them into two quantitative dimensions (Figure S1). The first dimension showed a high correlation both with the score of each muscle and with motor status (Spearman's rho correlation = 0.843, *p*‐value < 0.001), which highlights the potential use of this dimension as a proxy of global severity of intramuscular fatty replacement (Table S3).

**FIGURE 5:**
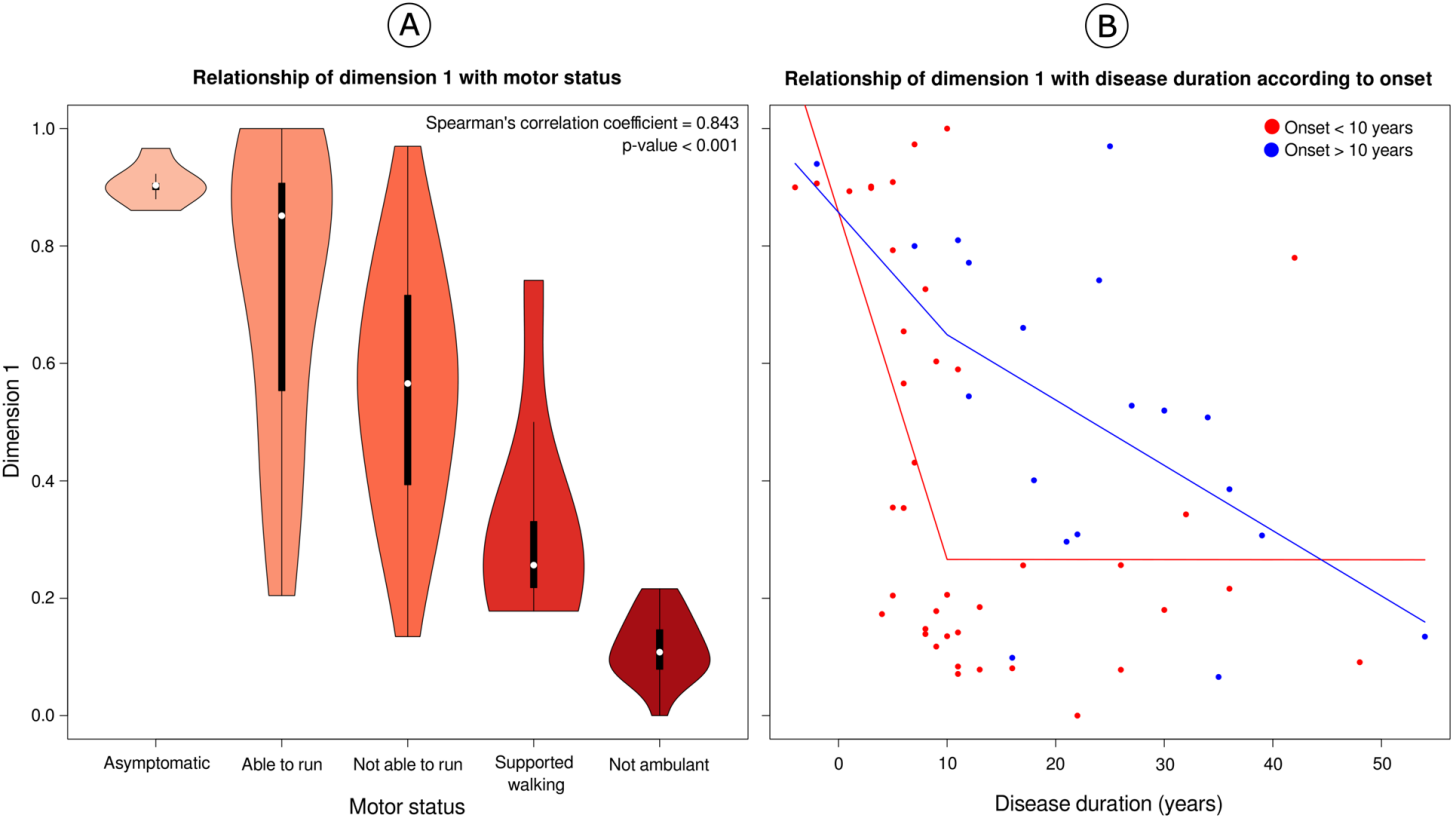
(A) Violin plots showing the distribution of dimension 1 according to the motor status at the time of the MRI. (B) Scatter Plot for dimension 1 according to disease duration. Patients with onset in the first decade are indicated in red while those with a later onset are plotted in blue. Lines represent the results of the prediction. The figure illustrates the cross‐sectional relationship between disease duration and the first MRI dimension. The dispersion of early‐onset cases (red dots) reflects heterogeneous severity at comparable durations, consistent with variable individual trajectories.

We also modeled the impact of disease onset on the relationship between disease duration and global intramuscular fatty replacement using several approaches. Given the non‐linear relationship between disease duration and the first MRI summary dimension, we fitted linear‐spline models. Several knot positions were compared using adjusted *R* [2], residual diagnostics, leverage statistics, and 10‐fold cross‐validated RMSE (Figure S2). A knot at 10 years provided the best overall performance and was consistent with the clinically recognized acceleration of decline during the first decade of disease evolution. Linear splines showed a better performance ($R_{\mathrm{adj}}^{2}=0.362\;\mathrm{vs}.R_{\mathrm{adj}}^{2}=0.192$); (Table S3) and supported a non‐linear relationship between disease duration and the first dimension of intramuscular fatty replacement. Age at disease onset modulated this relationship, with a steeper slope observed in the first 10 years of age for early‐onset patients, suggesting faster progression in the first decade, while later‐onset patients experienced a more linear decline (Figure 5). Notably, the wide dispersion observed among early‐onset patients indicates substantial inter‐individual variability in the extent of MRI abnormalities for similar disease durations, suggesting that rates of progression may differ across individuals. Disease duration was also significantly correlated with increased signal abnormalities representative of intramuscular fatty replacement, modulated by disease onset in the different muscles of the scapular girdle, trunk, pelvic girdle and thigh (Figure S7).

## 4 Discussion

This study contributes to the comprehensive characterization of the selective pattern of muscle pathology in sarcoglycanopathies imaged by MRI, with a specific focus on UB segments. As in LB, homogeneous results were observed indistinctly of the defective sarcoglycan gene. Further analysis of the variability of fatty replacement within single muscles strengthened the correlation between the degree of changes and deterioration of motor function as well as disease duration, and its modulation by age at disease onset. Finally, we confirmed that gradients of involvement, both within entire muscles and across different regions of the body, exist and are clear hallmarks of LGMDR3‐R6.

Although sarcoglycanopathies generally display a predominant involvement of the pelvic girdle region, selected muscles from the UB are also progressively involved in the initial stages of the disease. Therefore, concomitantly with the early involvement detected in the adductor magnus, long head of biceps femoris, gluteus maximus, and lumbar paravertebral muscles, latissimus dorsi and subscapularis also become affected. Our cross‐sectional analysis suggests that the latissimus dorsi is among the earliest and most consistently affected upper‐body muscles, particularly in mildly symptomatic patients. Later on, subscapularis, serratus anterior, trapezius, levator scapulae, supraspinatus, and infraspinatus muscles show signs of fat replacement while muscles from the head, neck, and forearms are widely preserved, even at very late stages. Thoracic paravertebral muscles are also mostly spared or mildly affected throughout the disease course.

Selective involvement of UB muscles has been described in various myopathies [7, 11]. The description of upper‐body patterns in sarcoglycanopathies may assist in the differential diagnosis of clinically overlapping disorders such as late‐onset Pompe disease, dystrophinopathies, and other LGMDs. In fact, several of these myopathies may share similar patterns in the LB (Table 1 [11, 15, 23, 24, 25, 26, 27, 28, 29, 30, 31, 32]), making our findings more remarkable. Pompe disease, for instance, is often characterized by sparing of the lower leg, which is otherwise typical of sarcoglycanopathies, yet can be distinguished by the early and severe involvement of the tongue, which is instead spared in sarcoglycanopathies. Although no direct comparative data were included in this study, the early involvement of the latissimus dorsi and subscapularis with relative preservation of the arm and forearm muscles differs from the imaging patterns described in dystrophinopathies (e.g., Becker and Duchenne muscular dystrophy) [15, 25, 26] and calpainopathy [27].

**TABLE 1:** Differential diagnosis of sarcoglycanopathies on whole‐body imaging.

	Similarities with sarcoglycanopathies on LB MRI	Distinctness of sarcoglycanopathies on UB MRI
Late‐onset Pompe disease [11, 23, 24]	Greater involvement of gluteus medius and minimus than maximus. Relative or complete lower leg sparing	Sparing of the tongue
Dystrophinopathies [15, 25, 26]	Early involvement of glutei, biceps femoris and vastus lateralis, with calf hypertrophy and sparing of sartorius and gracilis	Early involvement of latissimus dorsi. Greater involvement of the periscapular than arm muscles
LGMDR1/2A calpain‐3‐related [27]	Severe involvement of the posterior compartment of the thigh and glutei muscles	Relative preservation of anterior compartment of the arms
LGMDR2/2B dysferlin‐related [28, 29, 30]	Involvement of posterior thigh muscles	Relative sparing of the arm and forearm musculature
LGMDR23/Laminin α2‐related muscular dystrophy [31]	Greater involvement of gluteus medius and minimus than maximus, posterior thigh muscle involvement	Absence of brain white matter involvement. Sparing of temporalis muscle
LGMDD1/1D DNAJB6‐related [32]	Early involvement of posterior compartment of the thigh, with relative quadriceps sparing compared to posterior thigh	More severe involvement of the periscapular muscles and trapezius

*Note:* Myopathies exhibiting clinical symptoms and lower body MRI (LB MRI) patterns resembling those found in sarcoglycanopathies, can be distinguished by the unique upper body MRI patterns (UB MRI).

As generally expected in muscular dystrophies, the extent of intramuscular fat replacement in sarcoglycanopathies seems tightly linked with disease progression and functional impairment. Using dimensionality reduction techniques and linear splines, we showed in a large cohort of patients across a wide spectrum of severities that the effect of disease progression is modulated by age of disease onset. Patients with early onset present a more rapid, generalized muscle degeneration in their first decade of life, while patients with later onset show a more linear, slower decline. This behavior is similar in the different body regions and in the individual muscles and is in line with the currently available cross‐sectional clinical data [4]. Beyond these group‐level trends, the considerable variability observed among early‐onset patients for similar disease durations (Figure 5) indicates that the course of degeneration is not uniform, underscoring the heterogeneity that characterizes the disease. This variability is highly relevant for refining our understanding of the natural history and for informing trial design, particularly regarding patient stratification and expected progression windows. However, it should be noted that our observations are based on cross‐sectional data and therefore reflect associations between disease duration and MRI abnormalities rather than direct longitudinal progression.

**FIGURE 6:**
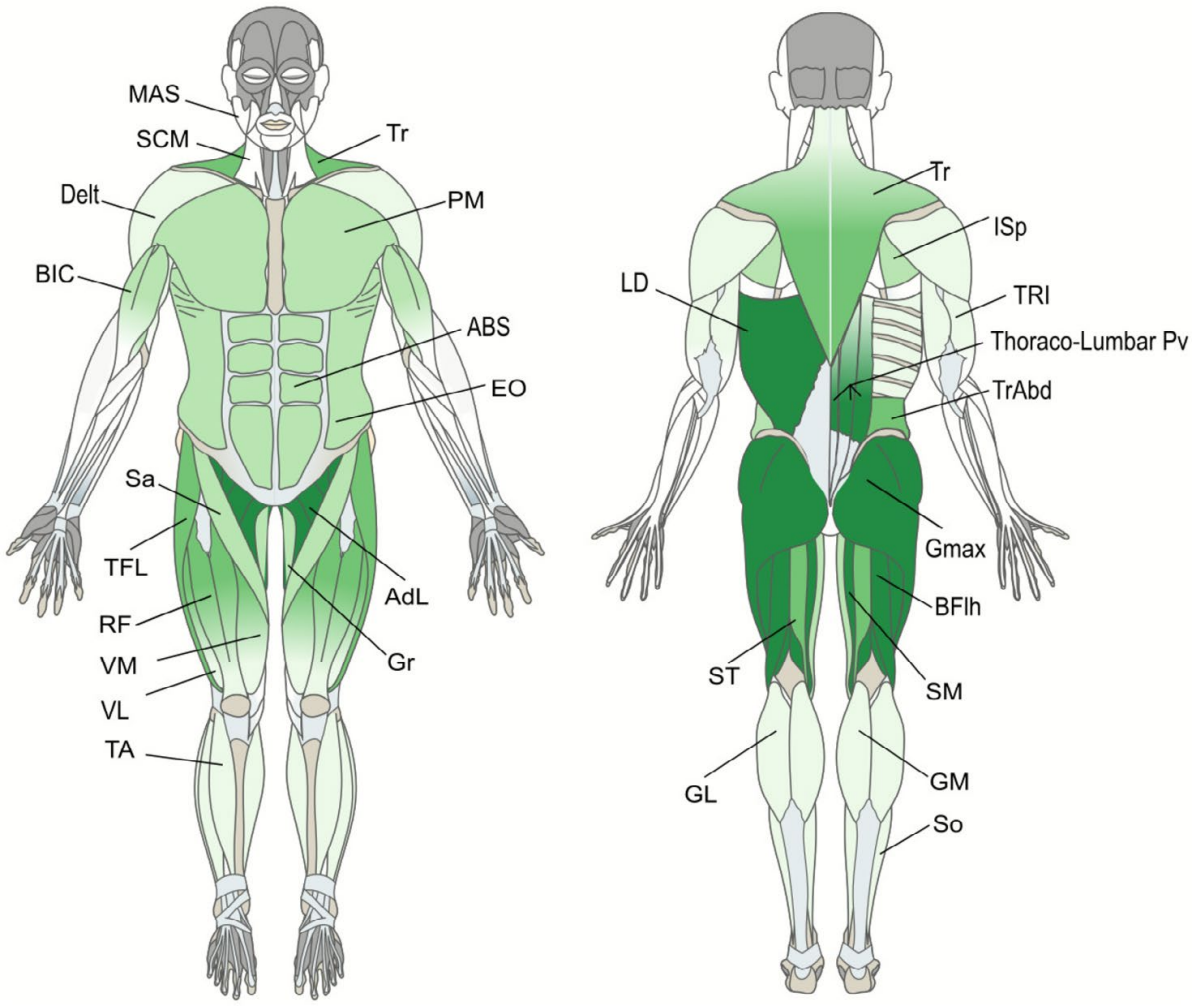
Visual representation of the whole‐body pattern of muscle degeneration in sarcoglycanopathies. Darker color corresponds to more severe involvement. Gradients in trapezius, arm muscles and quadriceps are also represented. Body muscles not investigated in the current study are marked in gray. MAS (masseter); SCM (sternocleidomastoid); Delt (deltoid); BIC (biceps brachii); Sa (sartorius); TFL (tensor fasciae latae); RF (rectus femoris); VM (vastus medialis); VL (vastus lateralis); TA (tibialis anterior); Tr (trapezius); PM (pectoralis major); ABS (rectus abdominis); EO (external obliquus abdominis); AdL (adductor longus); Gr (gracilis); ST (semitendinosus); GL (gastrocnemius lateralis); Isp (infraspinatus); TRI (triceps brachii); Gmax (gluteus maximus); BFlh (long head biceps femoris); SM (semimembranosus); GM (gastrocnemius medialis); So (soleus).

The WB distribution of muscle degeneration shows distinctive features in sarcoglycanopathies (Figure 6). We detected a striking involvement of proximal limb muscles with preservation of distal muscles until late phases of progression, with degeneration progressing in a rather synchronized and, to some extent, predictable way in the scapular and pelvic girdles and thighs, thus configuring a real paradigm of “limb‐girdle muscular dystrophy”. Gradients of fat replacement were consistently identified in different muscles and muscle compartments. These gradients are observed both intramuscularly (e.g., within the vastus lateralis [10]) and globally (e.g., in the lower limb, thigh muscles are more affected than distal lower leg ones). We have demonstrated that these gradients are reproducibly observed in UB regions as well, where they either follow the body axis caudo‐cranially, with progressive degeneration of paraspinal and trapezius muscles and sparing of intracranial muscles, or the limb axis in a proximo‐distal direction, both regionally (arm more affected than forearm) and intramuscularly (“descending” involvement in biceps and triceps brachii). Although a distribution of damage following the body or muscle axes is not a prerogative of sarcoglycanopathies and can also be seen in other disorders such as Facioscapulohumeral muscular dystrophy type 2 versus type 1 [33] and inclusion body myositis with preferential distal anterior thigh involvement [34], the particular gradients described here seem to be specific to LGMDR3‐R6. It is intriguing and one can speculate on the possible biological explanations of this phenomenon, which might implicate embryological development [35, 36] different abundance of protein or transcript levels in different muscles and different muscle regions [37], or even different cellular and molecular compositions of the extracellular matrix in different muscle portions [38].

Remarkably, the involvement of scapular girdle muscles such as the latissimus dorsi, subscapularis, and serratus anterior occurring in the early phases of the disease can have implications on daily living activities. The latissimus dorsi plays a crucial role in shoulder stabilization during various movements such as arm extension, adduction, and internal rotation, and the early involvement may impair actions like pulling, lifting, or reaching objects behind the body [39]. The main action of the subscapularis is also shoulder stabilization, with a contribution to internal rotation of the arm: it is activated in actions such as throwing, pushing, and reaching across the body. However, the latissimus dorsi and subscapularis have synergistic actions with other muscles in the scapular girdle, which are involved in later phases of the disease [39]. Compensation by these other synergistic muscles could be an explanation for the absence of significant upper limb weakness in early disease. Nonetheless, targeted upper limb scales and strength tests could be able to detect subtle impairment and its impact on daily activities, particularly in younger patients.

Our study has limitations, primarily related to its retrospective and multicentric design. Most MRI datasets included only T1‐weighted whole‐body sequences, and acquisition protocols were not standardized across centers. The lack of a unified protocol may have reduced the precision of assessments in long and thin muscles of the upper girdle, neck, and arms [14] and the absence of a standardized clinical evaluation protocol—including upper‐limb functional scales—limited the integration of imaging and clinical measures. The scarcity of available data on UB muscle MRI, especially in children with other muscular diseases, also hindered a thorough comparison. These constraints underscore the need for future prospective studies using harmonized Dixon or other quantitative protocols, which will be essential to complement the semi‐quantitative scoring used here and to support the development of imaging biomarkers for clinical trials. Importantly, by providing a detailed whole‐body map of selective muscle involvement and identifying the most consistently affected muscle groups and gradients, our study offers a robust framework for designing future studies and defining optimal regions of interest for quantitative analyses [13, 40]. Another partial limitation of this study is the unbalanced distribution of sarcoglycan subtypes, with a predominance of LGMDR3 compared to LGMDR4‐6, reflecting the relative prevalence of these genotypes in Europe and South America [4, 41]. This could have limited the power to identify subtle genotype‐specific differences. Therefore, even if our analysis strongly suggests that there is a common imaging phenotype shared across sarcoglycanopathy subtypes, future multicenter efforts including larger, more balanced cohorts will be required to explore potential differences in patterns and progression course.

In conclusion, the study results defined the topography of muscle involvement in disorders caused by mutations in one of the four sarcoglycan genes, which are characterized by a similar and consistent pattern on WB imaging and peculiar gradients of fat replacement along the body and limb axis, with the cranial and distal limb muscles being the most resistant to pathology. Specific UB muscles, such as the latissimus dorsi and subscapularis, are affected at the same time as those in the LB, highlighting an early UB involvement in sarcoglycanopathies warranting further in‐depth clinical investigations. Finally, the extent of fat replacement, especially in the most affected body regions, significantly correlated with disease duration. This correlation was impacted by age at disease onset, which has clear implications for disease monitoring and prognostication.

## Author Contributions

All authors contributed to the acquisition and analysis of data. L.C.‐C., F.M., D.G.‐A., and G.T. contributed to the conception and design of the study. L.C.‐C., D.G.‐A., and G.T. contributed to drafting a significant portion of the manuscript and preparing the figures. All authors approved the final version of the manuscript.

## Funding

This work was supported by Comisión Nacional de Investigación Científica y Tecnológica, FONDECYT 1151383. NIHR Newcastle Biomedical Research Centre.

## Conflicts of Interest

The authors declare no conflicts of interest.

## Supporting information


**Data S1:** acn370303‐sup‐0001‐Supinfo.pdf.

## Data Availability

MRI scores are openly available in Figshare at doi: 10.6084/m9.figshare.25481632. Raw imaging data supporting the findings of this study are available on request to the corresponding author.
