# Supplementary material for: Whole‐Body Pattern of Muscle Degeneration and Progression in Sarcoglycanopathies
Source: Ann Clin Transl Neurol. 2025 Dec 31;13(6):1129–40. doi: 10.1002/acn3.70303 (PMC13251455; doi:10.1002/acn3.70303)
Supplement: Supplementary file 1 — Data S1: acn370303‐sup‐0001‐Supinfo.pdf. [file ACN3-13-1129-s001.pdf]

Supplementary material

Table S1 Clinical features

CODE	COUNTRY	SEX	GENE	MRI TYPE	AGE AT MRI (y)	MOTOR STATUS AT MRI	AGE AT ONSET (y)	SIGNS AND SYMPTOMS AT ONSET
P1	Argentina	M	SGCA	WBMRI	16	supported walking	7	Walking difficulties
P2	Argentina	M	SGCA	WBMRI	13	supported walking	8	Walking difficulties
P3	Belgium	M	SGCA	WBMRI	51	not able to run	33	Difficulties in running and climbing stairs
P4	Belgium	F	SGCA	WBMRI	48	not able to run	Childhood	Difficulties in running and climbing stairs
P5	Belgium	F	SGCA	WBMRI	38	not able to run	Childhood	Difficulties in running and climbing stairs
P6	Chile	F	SGCB	WBMRI	29	non ambulant	13	Lower limb weakness
P7	Chile	F	SGCA	WBMRI	32	non ambulant	10	Lower limb weakness
P8	Germany	M	SGCA	UB&LBMRI	11	not able to run	2	Difficulties in running
P9	Germany	F	SGCA	UB&LBMRI	21	supported walking	4	Clumsiness
P10	Denmark	M	SGCG	WBMRI	46	non ambulant	10	Walking difficulties
P11	Denmark	F	SGCA	WBMRI	21	non ambulant	10	Difficulties in running and climbing stairs
P12	United Kingdom	M	SGCG	UB&LBMRI	17	non ambulant	6	Walking difficulties
P13	Spain	M	SGCA	WBMRI	36	supported walking	6	Difficulties in running
P14	Spain	F	SGCA	WBMRI	34	supported walking	8	Post-exercise muscle pain
P15	Spain	F	SGCA	WBMRI	34	not able to run	22	Myalgia
P16	Spain	F	SGCA	WBMRI	49	not able to run	Second decade	Proximal weakness
P17	Spain	F	SGCA	WBMRI	47	not able to run	40	Proximal and axial weakness
P18	Spain	F	SGCA	WBMRI	51	not able to run	Second decade	Proximal lower limb weakness
P19	Spain	M	SGCA	WBMRI	47	not able to run	35	Proximal weakness
P20	Spain	M	SGCB	WBMRI	33	not able to run	11	Exercise intolerance
P21	Spain	F	SGCG	WBMRI	16	non ambulant	8	Myalgia
P22	Spain	F	SGCG	WBMRI	4	asymptomatic	8	Difficulties in running
P23	Spain	M	SGCG	WBMRI	7	asymptomatic	/	Asymptomatic hyperCKemia

P24	Spain	M	SGCG	WBMRI	6	able to run	3	Clumsiness
P25	Spain	M	SGCG	WBMRI	7	able to run	6	Easy fatigability
P26	Spain	M	SGCA	WBMRI	5	asymptomatic	7	Difficulties in running
P27	Spain	M	SGCA	WBMRI	9	asymptomatic	/	Asymptomatic hyperCKemia
P28	Spain	M	SGCB	WBMRI	15	non ambulant	2	Development delay
P29	France	F	SGCB	WBMRI	16	non ambulant	6	Walking difficulties
P30	France	M	SGCA	WBMRI	10	able to run	4	Difficulties in climbing stairs
P31	Italy	M	SGCA	UB&LBMRI	34	not able to run	13	Difficulties in running
P32	Italy	F	SGCG	UB&LBMRI	50	non ambulant	15	Difficulties in climbing stairs
P33	Italy	F	SGCA	UB&LBMRI	29	not able to run	12	Myalgia
P34	Italy	F	SGCA	UB&LBMRI	10	asymptomatic	/	Asymptomatic hyperCKemia
P35	Italy	F	SGCA	UB&LBMRI	54	not able to run	45?	Asymptomatic hyperCKemia
P36	Italy	M	SGCA	UB&LBMRI	50	not able to run	23	Difficulties in climbing stairs
P37	Italy	M	SGCA	UB&LBMRI	41	able to run	11	Myalgia
P38	Italy	F	SGCA	UB&LBMRI	47	not able to run	22	Lower limbs weakness
P39	Italy	M	SGCA	UB&LBMRI	62	supported walking	38	Difficulties in climbing stairs
P40	Italy	M	SGCA	UB&LBMRI	67	not able to run	13	Difficulties in climbing stairs
P41	Italy	F	SGCB	UB&LBMRI	20	not able to run	9	Difficulties in running
P42	Italy	M	SGCD	UB&LBMRI	12	non ambulant	4	Difficulties in running and climbing stairs
P43	Italy	M	SGCA	UB&LBMRI	11	able to run	3	Toe walking
P44	Italy	M	SGCB	UB&LBMRI	16	non ambulant	3	Walking difficulties
P45	Italy	F	SGCA	UB&LBMRI	16	able to run	9	Myalgia
P46	Italy	M	SGCB	UB&LBMRI	8	not able to run	2	Walking difficulties
P47	Italy	F	SGCA	UB&LBMRI	10	able to run	3	Proximal weakness
P48	Italy	M	SGCB	UB&LBMRI	9	able to run	4	Walking difficulties
P49	Italy	F	SGCG	UB&LBMRI	32	non ambulant	6	Proximal lower limb weakness
P50	Italy	F	SGCA	UB&LBMRI	49	able to run	38	Post-exercise muscle pain
P51	Italy	F	SGCG	UB&LBMRI	16	able to run	6	Myalgia

P52	Italy	F	SGCB	UB&LBMRI	14	non ambulant	5	Difficulties in running
P53	Italy	F	SGCA	UB&LBMRI	59	supported walking	20	Walking difficulties
P54	Italy	M	SGCA	UB&LBMRI	57	able to run	59	Muscle cramps and easy fatigability
P55	Italy	M	SGCA	UB&LBMRI	27	asymptomatic	/	Asymptomatic hyperCKemia
P56	The Netherlands	M	SGCG	UB&LBMRI	22	non ambulant	6	Walking difficulties
P57	The Netherlands	M	SGCG	UB&LBMRI	18	non ambulant	7	Walking difficulties
P58	Russia	M	SGCA	WBMRI	10	non ambulant	6	Difficulties in climbing stairs
P59	Russia	M	SGCA	WBMRI	13	non ambulant	3	Toe walking
P60	Russia	F	SGCA	WBMRI	8	able to run	5	Toe walking
P61	Russia	F	SGCA	WBMRI	8	not able to run	3	Myalgia
P62	Russia	M	SGCA	WBMRI	12	able to run	7	Difficulties in running and climbing stairs
P63	Russia	M	SGCA	WBMRI	9	able to run	3	Myalgia
P64	Slovenia	F	SGCA	UB&LBMRI	55	non ambulant	7	Difficulties in running

Sex: F, female; M, male. MRI type: UB&LBMRI, Upper body and lower body MRI; WBMRI, Whole body MRI. y, years of age.

Table S2 Genetic data

ID	Sex	GENE	Genotype	cDNA[a]	Protein	MutationType	Reported in LOVD	ACMG criteria	Reduced sarcoglycan staining[b]	ACMG Subcriteria	gnomAD	Publication[c]
P1	M	SGCA	CHET	c.161delT	p.Val54Alafs*157	Frameshift	Pathogenic	Pathogenic	Yes	PVS1, PM2, PP5	not found	
				c.796G>A	p.Asp266Asn	Missense	-	Uncertain significance		PM2, PP2, BP4	not found	-
P2	M	SGCA	CHET	c.161delT	p.Val54Alafs*157	Frameshift	Pathogenic	Pathogenic	Yes	PVS1, PM2, PP5	not found	
				c.796G>A	p.Asp266Asn	Missense	-	Uncertain significance		PM2, PP2, BP4	not found	-
P3	M	SGCA	CHET	c.229C>T	p.Arg77Cys	Missense	Pathogenic	Pathogenic		PP5, PM1, PM2, PP2, PP3	0.00045	
				c.739G>A	p.Val247Met	Missense	Pathogenic	Pathogenic		PP5, PM1, PP2, PP3	0.00011	
P4	F	SGCA	CHET	c.229C>T	p.Arg77Cys	Missense	Pathogenic	Pathogenic		PP5, PM1, PM2, PP2, PP3	0.00045	
				c.739G>A	p.Val247Met	Missense	Pathogenic	Pathogenic		PP5, PM1, PP2, PP3	0.00011	
P5	F	SGCA	H	c.739G>A	p.Val247Met	Missense	Pathogenic	Pathogenic		PP5, PM1, PP2, PP3	0.00011	
				c.739G>A	p.Val247Met	Missense	Pathogenic	Pathogenic		PP5, PM1, PP2, PP3	0.00011	
P6	F	SGCB	H	c.621+1G>A	p.?	Splicing variant	Pathogenic	Pathogenic	Yes	PVS1, PM2, PP4_mod, PP5	not found	DOI: 10.3390/genes13061076
				c.621+1G>A	p.?	Splicing variant	Pathogenic	Pathogenic		PVS1, PM2, PP4_mod, PP5	not found	
P7	F	SGCA	H	c.746T>C	p.Leu249Pro	Missense	Pathogenic	Pathogenic	Yes	PM1, PM2, PP3	not found	DOI: 10.3390/genes13061076

				c.229C>T	p.Arg77Cys	Missense	Pathogenic	Pathogenic		PP5, PM1, PM2, PP2, PP3	0.00045	
P8	M	SGCA	CHET	c.100C>T	p.Arg34Cys	Missense	Pathogenic	Pathogenic		PP5, PM2, PM5, PP2, PP3	not found	
				c.229C>T	p.Arg77Cys	Missense	Pathogenic	Pathogenic		PP5, PM1, PM2, PP2, PP3	0.00045	
P9	F	SGCA	H	c.229C>T	p.Arg77Cys	Missense	Pathogenic	Pathogenic		PP5, PM1, PM2, PP2, PP3	0.00045	
				c.229C>T	p.Arg77Cys	Missense	Pathogenic	Pathogenic		PP5, PM1, PM2, PP2, PP3	0.00045	
P10	M	SGCG	CHET	c.205G>C	p.Gly69Arg	Missense	Pathogenic	Likely pathogenic		PM2, PM5, PP3, PP5, BP1	not found	-
				c.385G>A	p.Gly129Ser	Missense	Uncertain significance	Pathogenic		PM2, PP3, PP2	0.00006	
P11	F	SGCA	H	c.229C>T	p.Arg77Cys	Missense	Pathogenic	Pathogenic		PP5, PM1, PM2, PP2, PP3	0.00045	
				c.229C>T	p.Arg77Cys	Missense	Pathogenic	Pathogenic		PP5, PM1, PM2, PP2, PP3	0.00045	
P12	M	SGCG	H	c.787G>A	p.Glu263Lys	Missense	Pathogenic	Likely pathogenic		PP5, PM2, PP3, BP1	0.00003	-
				c.787G>A	p.Glu263Lys	Missense	Pathogenic	Likely pathogenic		PP5, PM2, PP3, BP1	0.00003	-
P13	M	SGCA	CHET	c.307A>T	p.Ile103Phe	Missense	Pathogenic	Likely pathogenic	Yes	PM2, PM5, PP2, PP3	not found	-
				c.700G>A	p.Asp234Asn	Missense	Uncertain significance	Uncertain significance		PM2, PP2, BP4	0.00008	DOI: 10.1093/brain/awaa228

P14	F	SGCA	CHET	c.307A>T	p.Ile103Phe	Missense	Pathogenic	Likely pathogenic	Yes	PM2, PM5, PP2, PP3	not found	-
				c.700G>A	p.Asp234Asn	Missense	Uncertain significance	Uncertain significance		PM2, PP2, BP4	0.00008	DOI: 10.1093/brain/awaa228
P15	F	SGCA	CHET	c.724G>T	p.Val242Phe	Missense	Pathogenic	Likely pathogenic		PM2, PM5, PP5, PP2, PP3	not found	-
				c.850C>T	p.Arg284Cys	Missense	Pathogenic	Pathogenic		PP5, PM2, PP2, PP3	0.00014	
P16	F	SGCA	H	c.585-31_585-24delTCTGCTGA	p.Val196Trpfs*71	Non coding variant	Pathogenic	Uncertain significance	Yes	PM2, PP3	not found	DOI: 10.1016/j.nmd.2018.06.002
				c.585-31_585-24delTCTGCTGA	p.Val196Trpfs*71	Non coding variant	Pathogenic	Uncertain significance		PM2, PP3	not found	DOI: 10.1016/j.nmd.2018.06.002
P17	F	SGCA	H	c.585-31_585-24delTCTGCTGA	p.Val196Trpfs*71	Non coding variant	Pathogenic	Uncertain significance	Yes	PM2, PP3	not found	DOI: 10.1016/j.nmd.2018.06.002
				c.585-31_585-24delTCTGCTGA	p.Val196Trpfs*71	Non coding variant	Pathogenic	Uncertain significance		PM2, PP3	not found	DOI: 10.1016/j.nmd.2018.06.002
P18	F	SGCA	CHET	c.488dupG	p.Leu164Thrfs*27	Frameshift	Pathogenic	Pathogenic	Yes	PVS1, PM2, PP3	not found	
				c.661C>T	p.Arg221His	Missense	Pathogenic	Likely pathogenic		PP2, PP3, PM2	0.00006	DOI: 10.1371/journal.pone.0175343
P19	M	SGCA	H	c.585-31_585-24delTCTGCTGA	p.Val196Trpfs*71	Non coding variant	Pathogenic	Uncertain significance	Yes	PM2, PP3	not found	DOI: 10.1016/j.nmd.2018.06.002
				c.585-31_585-24delTCTGCTGA	p.Val196Trpfs*71	Non coding variant	Pathogenic	Uncertain significance		PM2, PP3	not found	DOI: 10.1016/j.nmd.2018.06.002
P20	M	SGCB	CHET	c.341C>T	p.Ser114Phe	Missense	Pathogenic	Pathogenic	Yes (brother affected)	PP5, PM2, PP3, BP1	0.00028	
				c.906_907insATGTTTGG	p.Gln303Metfs*32	Frameshift	-	Pathogenic		PVS2, PM2, PP3	not found	-
P21	F	SGCG	H	c.(578+1_579-1)_(702+1_703-1)del	p.fs*	Frameshift	Pathogenic	-	Yes	-	not found	DOI: 10.1038/ejhg.2008.9, 10.1002/humu.20829
				c.(578+1_579-1)_(702+1_703-1)del	p.fs*	Frameshift	Pathogenic	-		-	not found	DOI: 10.1038/ejhg.2008.9, 10.1002/humu.20829

P22	F	SGCG	H	c.525delT	p.Phe175Leufs*20	Frameshift	Pathogenic	Pathogenic		PVS1, PP5, PM2, PP3	not found	
				c.525delT	p.Phe175Leufs*20	Frameshift	Pathogenic	Pathogenic		PVS1, PP5, PM2, PP3	not found	
P23	M	SGCG	H	c.525delT	p.Phe175Leufs*20	Frameshift	Pathogenic	Pathogenic		PVS1, PP5, PM2, PP3	not found	
				c.525delT	p.Phe175Leufs*20	Frameshift	Pathogenic	Pathogenic		PVS1, PP5, PM2, PP3	not found	
P24	M	SGCG	H	c.848G>A	p.Cys283Tyr	Missense	Pathogenic	Pathogenic		PP5, PM1, PM2, PP3, BP1	not found	
				c.848G>A	p.Cys283Tyr	Missense	Pathogenic	Pathogenic		PP5, PM1, PM2, PP3, BP1	not found	
P25	M	SGCG	H	c.525delT	p.Phe175Leufs*20	Frameshift	Pathogenic	Pathogenic		PVS1, PP5, PM2, PP3	not found	
				c.525delT	p.Phe175Leufs*20	Frameshift	Pathogenic	Pathogenic		PVS1, PP5, PM2, PP3	not found	
P26	M	SGCA	CHET	c.574C>T	p.Arg192*	Nonsense	Pathogenic	Pathogenic		PVS1, PP5, PM2, PP3	0.00000574	
				c.850C>T	p.Arg284Cys	Missense	Pathogenic	Pathogenic		PP5, PM2, PP2, PP3	0.00014	
P27	M	SGCA	CHET	c.409G>A	p.Glu137Lys	Missense	Pathogenic	Pathogenic		PP5, PM2, PM5, PP2, PP3	not found	
				c.850C>T	p.Arg284Cys	Missense	Pathogenic	Pathogenic		PP5, PM2, PP2, PP3	0.00014	
P28	M	SGCB	H	c.82_86delGAGAG	p.Glu28Lysfs*5	Frameshift	Pathogenic	Pathogenic		PVS1, PM2, PP3, PP5	not found	
				c.82_86delGAGAG	p.Glu28Lysfs*5	Frameshift	Pathogenic	Pathogenic		PVS1, PM2, PP3, PP5	not found	
P29	F	SGCB	CHET	c.290G>A	p.Cys97Tyr	Missense	-	Uncertain significance	Yes	PM1, PM2, PP3	not found	-

				c.654_655delAA	p.Lys219Serfs*2	Frameshift	-	Pathogenic		PVS1, PM2, PP3, PP5	not found	
P30	M	SGCA	CHET	c.229C>T	p.Arg77Cys	Missense	Pathogenic	Pathogenic		PP5, PM1, PM2, PP2, PP3	0.00045	
				c.403C>T	p.Gln135*	Nonsense	Pathogenic	Pathogenic		PVS1, PM2, PP5, PP3	not found	
P31	M	SGCA	CHET	c.229C>T	p.Arg77Cys	Missense	Pathogenic	Pathogenic		PP5, PM1, PM2, PP2, PP3	0.00045	
				c.739G>A	p.Val247Met	Missense	Pathogenic	Pathogenic		PP5, PM1, PP2, PP3	0.00011	
P32	F	SGCG	H	c.525delT	p.Phe175Leufs*20	Frameshift	Pathogenic	Pathogenic		PVS1, PP5, PM2, PP3	not found	
				c.525delT	p.Phe175Leufs*20	Frameshift	Pathogenic	Pathogenic		PVS1, PP5, PM2, PP3	not found	
P33	F	SGCA	CHET	c.308 T>C	p.Ile103Thr	Missense	Pathogenic	Likely pathogenic		PM2, PP2, PP3, PP5	not found	DOI: 10.1056/NEJM199702273360904
				c.850C>T	p.Arg284Cys	Missense	Pathogenic	Pathogenic		PP5, PM2, PP2, PP3	0.00014	
P34	F	SGCA	CHET	c.229C>T	p.Arg77Cys	Missense	Pathogenic	Pathogenic		PP5, PM1, PM2, PP2, PP3	0.00045	
				c.739G>A	p.Val247Met	Missense	Pathogenic	Pathogenic		PP5, PM1, PP2, PP3	0.00011	
P35	F	SGCA	H	c.850C>T	p.Arg284Cys	Missense	Pathogenic	Pathogenic		PP5, PM2, PP2, PP3	0.00014	
				c.850C>T	p.Arg284Cys	Missense	Pathogenic	Pathogenic		PP5, PM2, PP2, PP3	0.00014	
P36	M	SGCA	CHET	c.403C>T	p.Gln135*	Nonsense	Pathogenic	Pathogenic		PVS1, PM2, PP5, PP3	not found	
				c.739G>A	p.Val247Met	Missense	Pathogenic	Pathogenic		PP5, PM1, PP2, PP3	0.00011	

P37	M	SGCA	CHET	c.229C>T	p.Arg77Cys	Missense	Pathogenic	Pathogenic	Yes	PP5, PM1, PM2, PP2, PP3	0.00045	
				c.346A>C	p.Thr116Pro	Missense	-	Uncertain significance		PM2, PP2, PP3	not found	-
P38	F	SGCA	CHET	c.229C>T	p.Arg77Cys	Missense	Pathogenic	Pathogenic		PP5, PM1, PM2, PP2, PP3	0.00045	
				c.403C>T	p.Gln135*	Nonsense	Pathogenic	Pathogenic		PVS1, PM2, PP5, PP3	not found	
P39	M	SGCA	CHET	c.229C>T	p.Arg77Cys	Missense	Pathogenic	Pathogenic		PP5, PM1, PM2, PP2, PP3	0.00045	
				c.739G>A	p.Val247Met	Missense	Pathogenic	Pathogenic		PP5, PM1, PP2, PP3	0.00011	
P40	M	SGCA	CHET	c.229C>T	p.Arg77Cys	Missense	Pathogenic	Pathogenic		PP5, PM1, PM2, PP2, PP3	0.00045	
				c.739G>A	p.Val247Met	Missense	Pathogenic	Pathogenic		PP5, PM1, PP2, PP3	0.00011	
P41	F	SGCB	H	c.265G>A	p.Val89Met	Missense	Pathogenic	Uncertain significance		PM2, PP3, PP5, PM2	0.00000795	PMID: 15938573; DOI: 10.1136/jnnp-2017-316736
				c.265G>A	p.Val89Met	Missense	Pathogenic	Uncertain significance		PM2, PP3, PP5, PM2	0.00000795	PMID: 15938573; DOI: 10.1136/jnnp-2017-316736
P42	M	SGCD	H	c.465_469delAGTGG	p.Glu155Aspfs*5	Frameshift	-	Likely pathogenic		PVS1, PM2	not found	-
				c.465_469delAGTGG	p.Glu155Aspfs*5	Frameshift	-	Likely pathogenic		PVS1, PM2	not found	-
P43	M	SGCA	H	c.92T>C	p.Leu31Pro	Missense	Pathogenic	Pathogenic		PP5, PS3, PM1, PM2, PP3	not found	
				c.92T>C	p.Leu31Pro	Missense	Pathogenic	Pathogenic		PP5, PS3, PM1, PM2, PP3	not found	

P44	M	SGCB	H	c.377_384dupCAGTAGGA	p.Gly129Glnfs*30	Frameshift	Pathogenic	Pathogenic		PVS1, PM2, PP3, PP5	0.00000398	
				c.377_384dupCAGTAGGA	p.Gly129Glnfs*30	Frameshift	Pathogenic	Pathogenic		PVS1, PM2, PP3, PP5	0.00000398	
P45	F	SGCA	H	c.739G>A	p.Val247Met	Missense	Pathogenic	Pathogenic		PP5, PM1, PP2, PP3	0.00011	
				c.739G>A	p.Val247Met	Missense	Pathogenic	Pathogenic		PP5, PM1, PP2, PP3	0.00011	
P46	M	SGCB	H	c.377_384dupCAGTAGGA	p.Gly129Glnfs*30	Frameshift	Pathogenic	Pathogenic		PVS1, PM2, PP3, PP5	0.00000398	
				c.377_384dupCAGTAGGA	p.Gly129Glnfs*30	Frameshift	Pathogenic	Pathogenic		PVS1, PM2, PP3, PP5	0.00000398	
P47	F	SGCA	CHET	c.271G>A	p.Gly91Ser	Missense	Pathogenic	Likely pathogenic		PM1, PM2, PM5, PP3	0.00000804	DOI: 10.1186/s12883-015-0428-8, 10.1002/humu.20642
				c.292 C>T	p.Arg98Cys	Missense	Pathogenic	Pathogenic		PP5, PM1, PM2, PM5, PP2, PP3	0.0000283	
P48	M	SGCB	H	c.377_384dupCAGTAGGA	p.Gly129Glnfs*30	Frameshift	Pathogenic	Pathogenic		PVS1, PM2, PP3, PP5	0.00000398	
				c.377_384dupCAGTAGGA	p.Gly129Glnfs*30	Frameshift	Pathogenic	Pathogenic		PVS1, PM2, PP3, PP5	0.00000398	
P49	F	SGCG	H	c.87dupT	p.Gly129Glnfs*30	Frameshift	Pathogenic	Pathogenic		PVS1, PM2, PP3, PP5	0.00000398	
				c.87dupT	p.Gly129Glnfs*30	Frameshift	Pathogenic	Pathogenic		PVS1, PM2, PP3, PP5	0.00000398	
P50	F	SGCA	H	c.739G>A	p.Val247Met	Missense	Pathogenic	Pathogenic		PP5, PM1, PP2, PP3	0.00011	
				c.739G>A	p.Val247Met	Missense	Pathogenic	Pathogenic		PP5, PM1, PP2, PP3	0.00011	
P51	F	SGCG	H	c.371G>A	p.Gly124Asp	Missense	Uncertain significance	Uncertain significance	Yes	PM2, PP3, BP1	not found	DOI: 10.1136/jnnp-2017-316736
				c.371G>A	p.Gly124Asp	Missense	Uncertain significance	Uncertain significance		PM2, PP3, BP1	not found	DOI: 10.1136/jnnp-2017-316736

P52	F	SGCB	H	c.552T>G	p.Tyr184*	Nonsense	Pathogenic	Pathogenic		PVS1, PM2, PP3, PP5	not found	
				c.552T>G	p.Tyr184*	Nonsense	Pathogenic	Pathogenic		PVS1, PM2, PP3, PP5	not found	
P53	F	SGCA	H	c.850C>T	p.Arg284Cys	Missense	Pathogenic	Pathogenic		PP5, PM2, PP2, PP3	0.000151	
				c.850C>T	p.Arg284Cys	Missense	Pathogenic	Pathogenic		PP5, PM2, PP2, PP3	0.000151	
P54	M	SGCA	H	c.850C>T	p.Arg284Cys	Missense	Pathogenic	Pathogenic		PP5, PM2, PP2, PP3	0.000151	
				c.850C>T	p.Arg284Cys	Missense	Pathogenic	Pathogenic		PP5, PM2, PP2, PP3	0.000151	
P55	M	SGCA	CHET	c.541C>T	p.Arg181Cys	Missense	Pathogenic	Uncertain significance		PM2, PP2, PB4	0.0000372	DOI: 10.1136/jmg.40.5.e67, 10.1016/j.pediatrneurol.2013.12.024
				c.86dupA	p.His29Glnfs*15	Frameshift	Pathogenic	Pathogenic		PVS1, PM2, PP5	not found	
P56	M	SGCG	H	c.505+2T>C	p.?	Splicing variant	Pathogenic	Pathogenic		PVS1, PM2, PP3, PP5	not found	
				c.505+2T>C	p.?	Splicing variant	Pathogenic	Pathogenic		PVS1, PM2, PP3, PP5	not found	
P57	M	SGCG	H	c.505+2T>C	p.?	Splicing variant	Pathogenic	Pathogenic		PVS1, PM2, PP3, PP5	not found	
				c.505+2T>C	p.?	Splicing variant	Pathogenic	Pathogenic		PVS1, PM2, PP3, PP5	not found	
P58	M	SGCA	H	c.271G>A	p.Gly91Ser	Missense	Pathogenic	Likely pathogenic		PM1, PM2, PM5, PP2, PP3	0.00000804	DOI: 10.1186/s12883-015-0428-8, 10.1002/humu.20642
				c.271G>A	p.Gly91Ser	Missense	Pathogenic	Likely pathogenic		PM1, PM2, PM5, PP2, PP3	0.00000804	DOI: 10.1186/s12883-015-0428-8, 10.1002/humu.20642
P59	M	SGCA	CHET	c.101G>A	p.Arg34His	Missense	Pathogenic	Pathogenic		PP5, PM2, PM5, PP2, PP3	0.0000239	

				c.20G>A	p.Trp7*	Nonsense	-	Pathogenic		PVS1, PM2, PP3	not found	
P60	F	SGCA	CHET	c.101G>A	p.Arg34His	Missense	Pathogenic	Pathogenic		PP5, PM2, PM5, PP2, PP3	0.0000239	
				c.20G>A	p.Trp7*	Nonsense	-	Pathogenic		PVS1, PM2, PP3	not found	
P61	F	SGCA	H	c.271G>A	p.Gly91Ser	Missense	Pathogenic	Likely pathogenic		PM1, PM2, PM5, PP2, PP3	0.00000804	DOI: 10.1186/s12883-015-0428-8, 10.1002/humu.20642
				c.271G>A	p.Gly91Ser	Missense	Pathogenic	Likely pathogenic		PM1, PM2, PM5, PP2, PP3	0.00000804	DOI: 10.1186/s12883-015-0428-8, 10.1002/humu.20642
P62	F	SGCA	CHET	c.229C>T	p.Arg77Cys	Missense	Pathogenic	Pathogenic		PP5, PM1, PM2, PP2, PP3	0.00045	
				c.402C>G	p.Tyr134*	Nonsense	Pathogenic	Pathogenic		PV1, PM2, PP3	not found	
P63	M	SGCA	CHET	c.271G>A	p.Gly91Ser	Missense	Pathogenic	Likely pathogenic		PM1, PM2, PM5, PP2, PP3	0.00000804	DOI: 10.1186/s12883-015-0428-8, 10.1002/humu.20642
				c.574C>T	p.Arg192*	Nonsense	Pathogenic	Pathogenic		PVS1, PP5, PM2, PP3	0.00000574	
P64	F	SGCA	H	c.229C>T	p.Arg77Cys	Missense	Pathogenic	Pathogenic		PP5, PM1, PM2, PP2, PP3	0.00045	
				c.229C>T	p.Arg77Cys	Missense	Pathogenic	Pathogenic		PP5, PM1, PM2, PP2, PP3	0.00045	

[a]cDNA transcript: SGCA: NM_000023; SGCG: NM_000231; SGCB: NM_000232; SGCD: NM_000337

[b]Only reported if they have a muscle biopsy

[c]Reported in case of a likely pathogenic or uncertain significance variant and a publication present (identifiers: DOI or PMID).

Abbreviations: ID: patient identifier. Sex: F, female; M, male. Gene: SGCA, α-sarcoglycan; SGCB, β-sarcoglycan; SGCD, δ-sarcoglycan; SGCG, γ-sarcoglycan. Genotype: CHET, Compound heterozygous; H, Homozygous. LOVD, Leiden Open Variation Database; ACMG, American College of Medical Genetics; ACMG subcriteria: PVS1, very strong evidence of pathogenicity; PS1-

4, Strong evidence of pathogenicity; PM1-6, Moderate evidence of pathogenicity; PP1-5, Supporting evidence of pathogenicity; BA1, Stand-Alone evidence of benign impact; BS1-4, Strong evidence of benign impact; BP1-7, Supporting evidence of benign impact. gnomAD, Genome Aggregation Database.(Richards et al., 2015)(Richards et al., 2015)(Richards et al., 2015)(Richards et al., 2015)

Figure S1

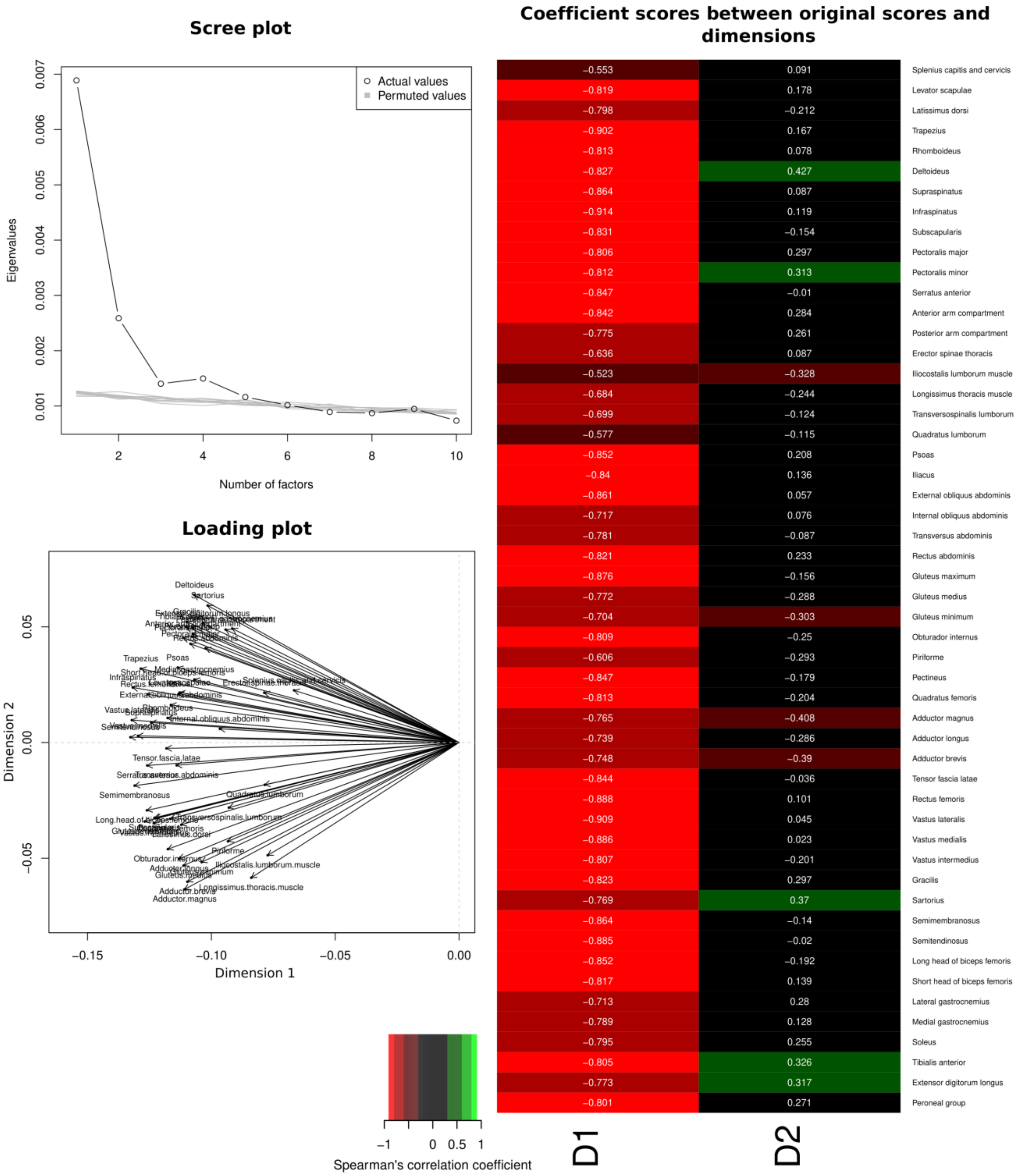

Figure S1 Multi-correspondence analysis with optimal scaling. Scree plot (upper left) indicating the eigenvalues for 10 pre-defined dimension reductions. Real data results were compared with results from 10-permutated databases. Only 2 dimensions reached a clear

difference with permutation data. Loading plot (bottom left) showing the loadings of original scores in the 2 dimensions of the final model. On the right, Spearman's rho correlation coefficients between the scores and the 2 dimensions. Strong negative correlations were found between the scores of most of the muscles and the dimension 1. In contrast, correlation between dimension 2 and muscle scores was low.

Figure S2:

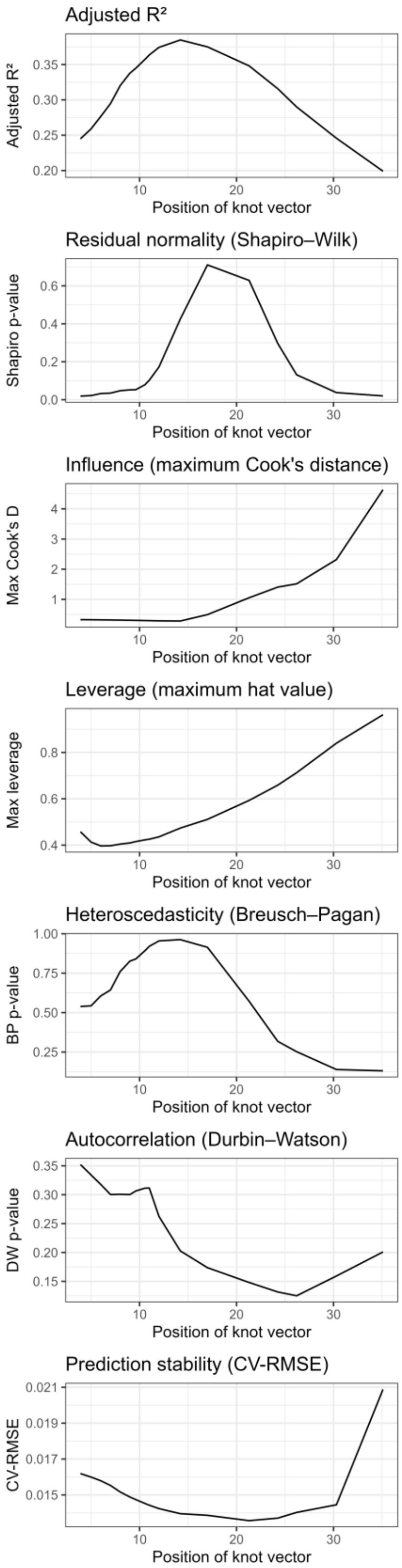

Figure S2 Diagnostic evaluation of spline knot placement. Seven complementary statistics were

computed for each candidate knot position: adjusted R^2 (model fit), Shapiro–Wilk p-value (residual normality), maximum Cook's distance (influential observations), maximum leverage (hat values), Breusch–Pagan p-value (heteroscedasticity), Durbin–Watson p-value (autocorrelation), and cross-validated RMSE (prediction stability). These metrics jointly assess goodness-of-fit, residual structure, robustness to influential data points, and generalisation performance. Knot position 10 consistently yields the optimal compromise, combining high fit, lower influence and leverage, stable CV-RMSE, and acceptable distributional assumptions, making it the most robust and reliable knot location.

Figure S3

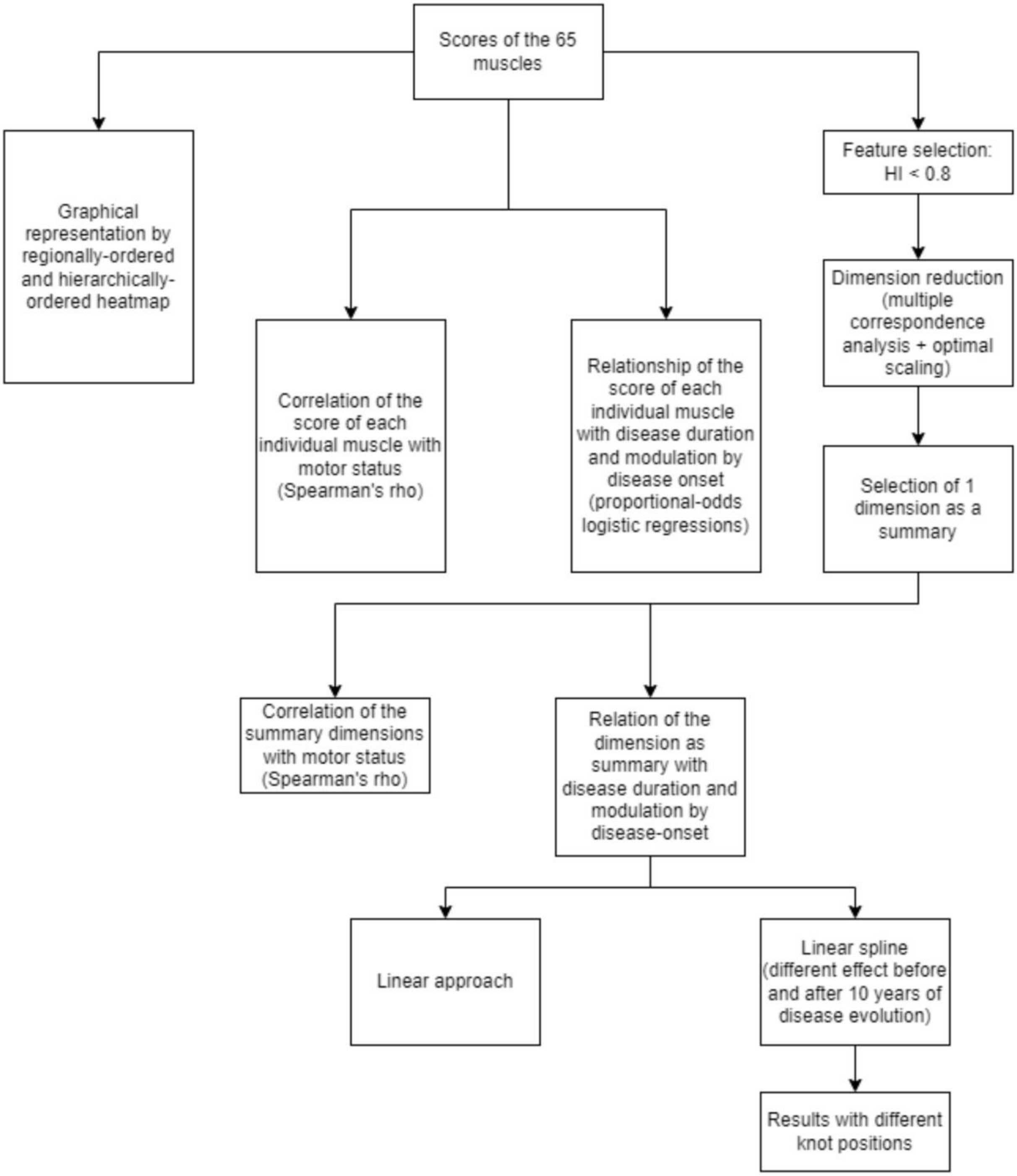

Figure S3 Data analysis process

Figure S4:

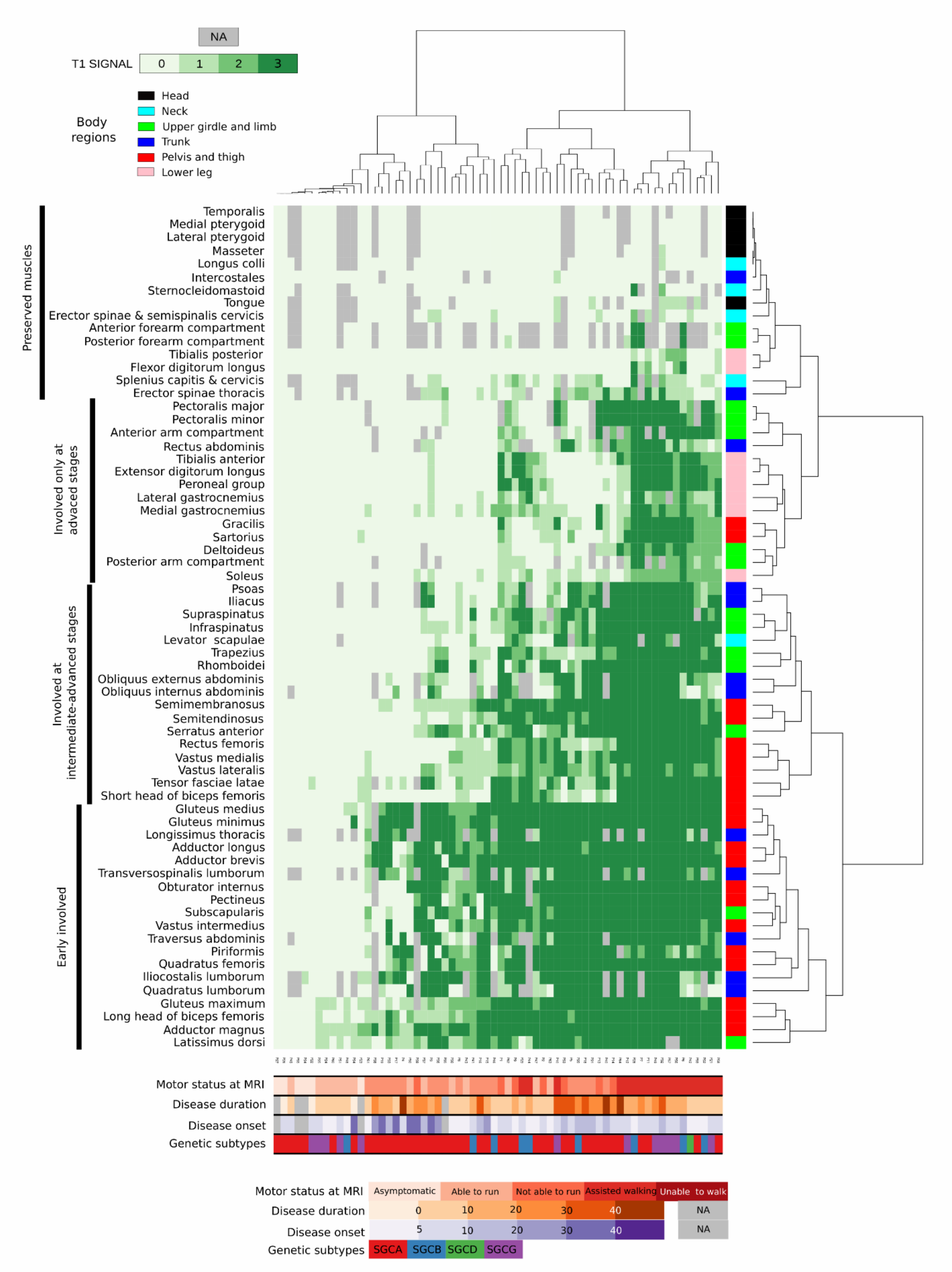

Figure S4 Hierarchical heatmap with muscles clustered according to degree of fat replacement. corresponding to the distances in the dendrogram on the right.

Figure S5:

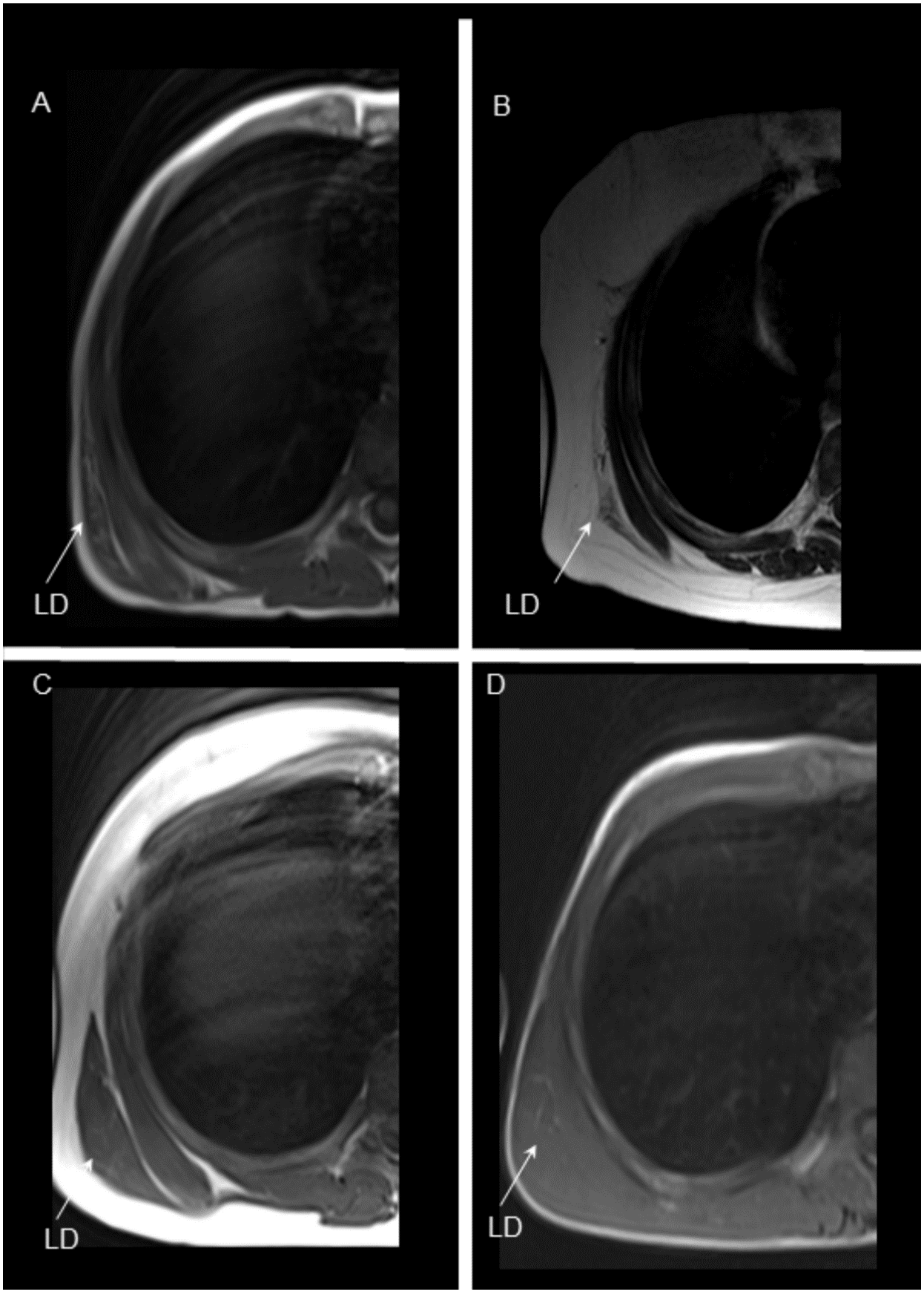

Figure S5 Early latissimus dorsi muscle (LD) fat infiltration. Axial T1w are shown. **(A)** P23, age 7 years, able to run, LGMDR5: the latissimus dorsi presents a mild involvement (Modified

Lamminen score of 1) compared to the preservation of the latissimus dorsi seen in **(C)**, asymptomatic patient with Becker Muscular Dystrophy, age 8 years. **(B)** P47, age 10 years, able to run, LGMDR3: latissimus dorsi presents a significant involvement with atrophy (Modified Lamminen score of 3) in contrast to preserved latissimus dorsi showed in an older (15 years of age asymptomatic patient with Becker Muscular Dystrophy **(D)**.

Figure S6:

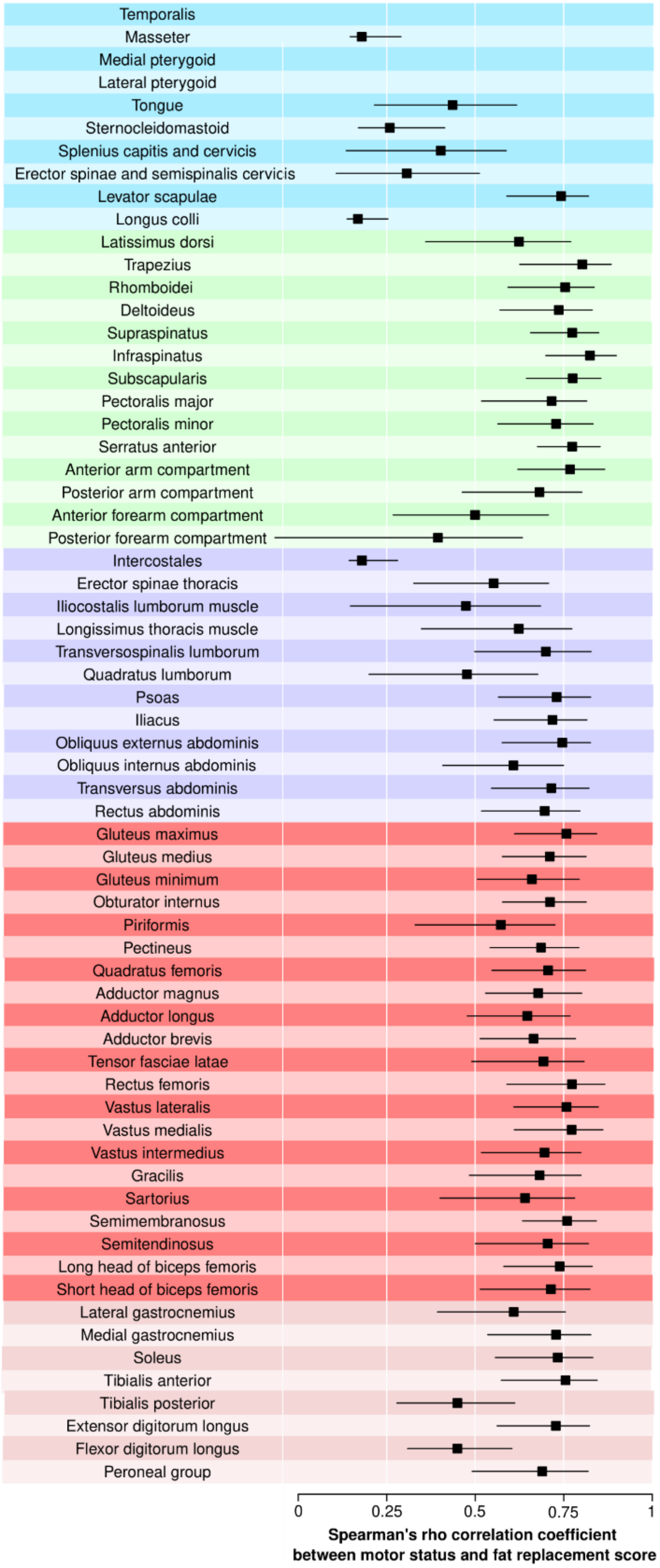
Temporalis
Masseter
Medial pterygoid
Lateral pterygoid
Tongue
Sternocleidomastoid
Splenius capitis and cervicis
Erector spinae and semispinalis cervicis
Levator scapulae
Longus colli
Latissimus dorsi
Trapezius
Rhomboidei
Deltoideus
Supraspinatus
Infraspinatus
Subscapularis
Pectoralis major
Pectoralis minor
Serratus anterior
Anterior arm compartment
Posterior arm compartment
Anterior forearm compartment
Posterior forearm compartment
Intercostales
Erector spinae thoracis
Iliocostalis lumborum muscle
Longissimus thoracis muscle
Transversospinalis lumborum
Quadratus lumborum
Psoas
Iliacus
Obliquus externus abdominis
Obliquus internus abdominis
Transversus abdominis
Rectus abdominis
Gluteus maximus
Gluteus medius
Gluteus minimum
Obturator internus
Piriformis
Pectineus
Quadratus femoris
Adductor magnus
Adductor longus
Adductor brevis
Tensor fasciae latae
Rectus femoris
Vastus lateralis
Vastus medialis
Vastus intermedius
Gracilis
Sartorius
Semimembranosus
Semitendinosus
Long head of biceps femoris
Short head of biceps femoris
Lateral gastrocnemius
Medial gastrocnemius
Soleus
Tibialis anterior
Tibialis posterior
Extensor digitorum longus
Flexor digitorum longus
Peroneal group
0
0.25
0.5
0.75
1
Spearman's rho correlation coefficient
between motor status and fat replacement score

Figure S6 Forest plot showing Spearman's rho correlation coefficient between motor status and fat replacement score for each muscle. Strong or moderate correlations were obtained for most muscles. Pelvic girdle, thigh, scapular girdle and arm muscles showed the highest correlation coefficients.

Table S3. Different models for evaluating disease duration effect on fat replacement dimension 1

	Linear model	Linear spline model with continuous age at onset	Linear spline model with age at onset split at 10 years
Adjusted R^2	0.192	0.362	0.347
Disease duration < 10 years	-0.0166 (p < 0.001)	-0.0668 (p < 0.001)	-0.0591 (p < 0.001)
Disease duration > 10 years	-0.0166 (p < 0.001)	-0.0007 (p=0.928)<br>Marginal: 0.0661 (p<0.001)	0.0001 (p=0.997)<br>Marginal: 0.0591<br>(p = 0.001)
Age at onset	Continuous	Continuous	Split at 10 years
Interaction term disease duration < 10 years	0.0006 (p = 0.012)	0.0015 (p = 0.004)	0.0383 (p=0.002)
Interaction term disease duration > 10 years	0.0006 (p = 0.012)	-0.0001 (p=0.774)<br>Marginal: -0.0016<br>(p=0.08)	-0.0111 (p=0.119)<br>Marginal: -0.04937<br>(p=0.007)

Figure S7:

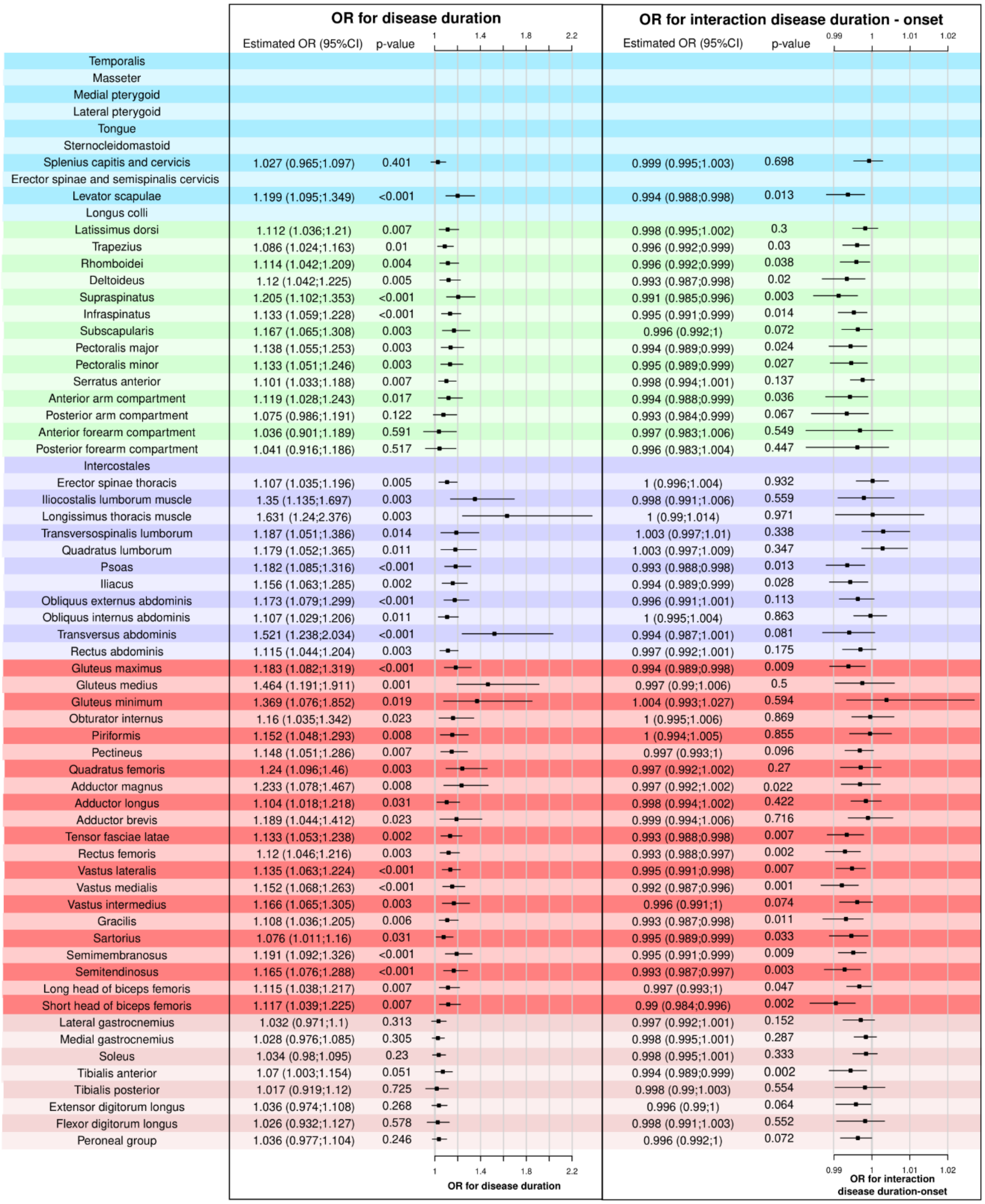

Figure S7 Forest plot showing the results of proportional-odds logistic regression. On the left, odds' ratios for disease duration in each muscle are shown. On the right, odds' ratios for the disease duration and disease onset are represented. The odds' ratios for disease duration in the scapular girdle, trunk, pelvic girdle and thigh regions were 1.1-1.2, with paravertebral lumbar muscles, gluteus maximus, quadratus femoris, adductor magnus, transversus abdominis and leg muscles

having lower odds ratios. The interaction term between disease duration and disease onset captures the modulatory effect of disease onset on the detrimental effect of disease duration.
